# 2D materials for conducting holes from grain boundaries in perovskite solar cells

**DOI:** 10.1038/s41377-021-00515-8

**Published:** 2021-03-31

**Authors:** Peng You, Guanqi Tang, Jiupeng Cao, Dong Shen, Tsz-Wai Ng, Zafer Hawash, Naixiang Wang, Chun-Ki Liu, Wei Lu, Qidong Tai, Yabing Qi, Chun-Sing Lee, Feng Yan

**Affiliations:** 1 Department of Applied Physics, The Hong Kong Polytechnic University, Hung Hom, Kowloon, Hong Kong China; 2 College of New Materials and New Energies, Shenzhen Technology University, 518118 Shenzhen, China; 3 Center of Super-Diamond and Advanced Films (COSDAF), Department of Chemistry, City University of Hong Kong, Hong Kong, China; 4 Energy Materials and Surface Sciences Unit (EMSSU), Okinawa Institute of Science and Technology Graduate University (OIST), 1919-1 Tancha, Onna-son, Okinawa, 904-0495 Japan; 5 University Research Facility in Materials Characterization and Device Fabrication, The Hong Kong Polytechnic University, Hung Hom, Kowloon, Hong Kong China

**Keywords:** Photonic devices, Solar energy and photovoltaic technology

## Abstract

Grain boundaries in organic–inorganic halide perovskite solar cells (PSCs) have been found to be detrimental to the photovoltaic performance of devices. Here, we develop a unique approach to overcome this problem by modifying the edges of perovskite grain boundaries with flakes of high-mobility two-dimensional (2D) materials via a convenient solution process. A synergistic effect between the 2D flakes and perovskite grain boundaries is observed for the first time, which can significantly enhance the performance of PSCs. We find that the 2D flakes can conduct holes from the grain boundaries to the hole transport layers in PSCs, thereby making hole channels in the grain boundaries of the devices. Hence, 2D flakes with high carrier mobilities and short distances to grain boundaries can induce a more pronounced performance enhancement of the devices. This work presents a cost-effective strategy for improving the performance of PSCs by using high-mobility 2D materials.

## Introduction

Perovskite solar cells (PSCs) based on organic–inorganic halide perovskites have been increasingly studied in recent years. Since the first report in 2009 of a perovskite material used in solar cells^1^, the power conversion efficiencies (PCEs) of PSCs have now reached a certified value higher than those of solar cells based on multi-crystalline Si, cadmium telluride or copper indium gallium diselenide, according to the efficiency chart provided by the National Renewable Energy Laboratory (NREL)^2^. Organic–inorganic halide perovskites have shown many advantages over conventional semiconductors for photovoltaics, including long carrier lifetimes, high light absorption, easy processing and low fabrication cost^3–11^. Therefore, PSCs are promising for use in practical applications in the future.

Grain boundaries (GBs) in PSCs have been found to be detrimental to the photovoltaic performance of devices^12^. Numerous papers have reported that defects in perovskite GBs should be passivated by suitable materials, such as quaternary ammonium halide^13^, fullerene derivatives^14–16^ and CH_3_NH_3_I (MAI)^17^, to alleviate carrier recombination and consequently improve device performance. Here, we report a novel method to overcome the drawback of perovskite GBs without passivating defects. Several 2D materials, including black phosphorus (BP), MoS_2_ and graphene oxide (GO), are specifically modified on the edge of perovskite GBs by a solution process. These 2D materials have high carrier mobilities, ultrathin thicknesses and smooth surfaces without dangling bonds^18–22^. The PCEs of the devices are substantially enhanced by the 2D flakes, in which BP flakes can induce the highest relative enhancement of ~15%. Notably, we find that under certain conditions, GBs modified with 2D materials are favourable for device performance. Therefore, a synergistic effect between 2D flakes and perovskite GBs is observed for the first time. Although nanotechnology using 2D materials in PSCs has been reported in some papers^20,23–26^, the synergistic effect between 2D flakes and perovskite GBs has not been reported until now. To better understand the underlying mechanism of the above effect, device simulation is conducted by using commercial software^27^. The hole conduction processes from GBs to 2D flakes in PSCs are clearly demonstrated, showing that the GBs and 2D flakes all act as hole channels in the devices. The simulation results confirm that the performance enhancement induced by BP is higher than that induced by the other 2D materials because BP has the highest hole mobility^28–34^. In addition, the modification of 2D flakes on the perovskite grains away from GBs has little effect on device performance, indicating that the synergistic effect of 2D flakes and perovskite GBs is essential to the performance enhancement in our devices.

## Results

### Modification of BP flakes on perovskite films

First, we modified the surfaces of CH_3_NH_3_PbI_3_ (MAPbI_3_) perovskite active layers in PSCs with BP flakes by a solution process. BP has recently emerged as a promising 2D semiconducting material for various applications owing to its tunable direct bandgap and high carrier mobility^28^. A BP dispersion solution was prepared using an ultrasonication method from BP crystals (Fig. S1a)^35^. Anhydrous isopropanol (IPA) was chosen as an orthogonal solvent for the BP dispersion to be coated on perovskite films. BP flakes were first coated on flat Si substrates and characterized with atomic force microscopy (AFM) (Fig. S1b). The average size and thickness of the BP flakes are estimated to be 39 ± 19 nm and 4.3 ± 2.0 nm, respectively (Fig. S1d, e). The high-resolution transmission electron microscopy (HRTEM) image of a BP flake confirms the orthorhombic crystal structure of BP (Fig. S2). Ultraviolet photoelectron emission spectra (UPS) of the BP thin films on silicon substrates were obtained (Fig. S3a). The valence band maximum (VBM) of BP is found to be ~−5.32 eV. Based on the PL spectrum of BP films (Fig. S4), the bandgap of BP flakes is estimated to be approximately 1.53 eV. Therefore, the conduction band minimum (CBM) of the BP flakes is calculated to be ~−3.79 eV.

**Fig. 1:**
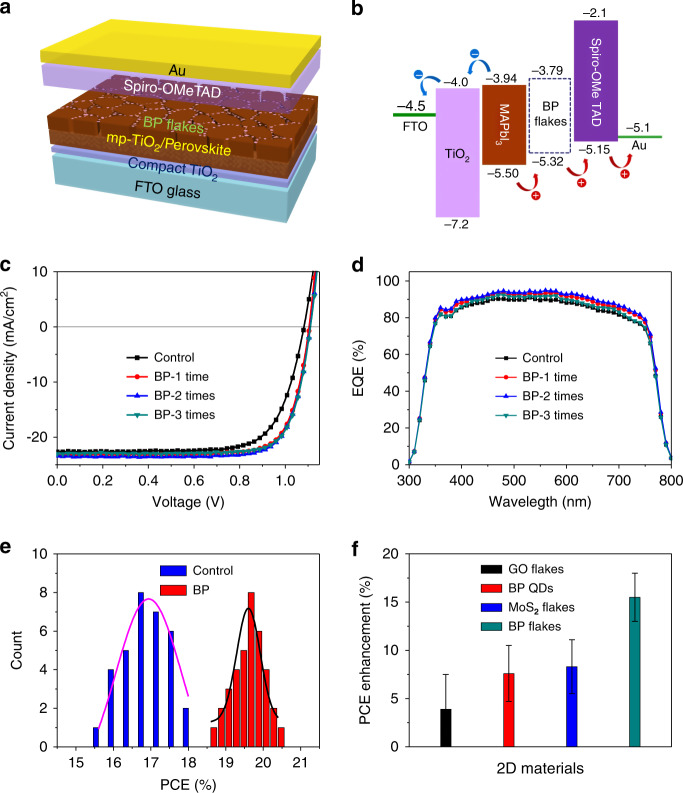
Device structure and photovoltaic performance of MAPbI_3_ PSCs. **a**, **b** Structural schematic diagram and energy-level band diagram of a BP-modified PSC with a normal structure. **c**, **d** Current density–voltage (*J–V*) curves (reverse scan) and external quantum efficiency (EQE) spectra of PSCs without (control) and with BP (1–3 times) deposited on the perovskite film surface. **e** Histogram of the power conversion efficiencies (PCEs) of PSCs without (control) and with the BP modification (2 coats of BP). The PCEs are derived from the reverse scans of the *J–V* curves. **f** Average PCE enhancement after the modification of perovskite films with different 2D materials. The error bar corresponds to the standard error for 30 devices

PSCs were fabricated with a device configuration of glass/fluorine-doped tin oxide (FTO)/compact TiO_2_ (c-TiO_2_)/mesoporous TiO_2_ (mp-TiO_2_)/MAPbI_3_/BP/2,2’,7,7’-tetrakis-(N,N-di-p-methoxyphenylamine)−9,9’-spirobifluorene (spiro-OMeTAD)/Au, as shown in Fig. 1a. MAPbI_3_ perovskite films were prepared by an anti-solvent-assisted crystallization method^36^. BP flakes were modified on perovskite layers by spin coating the BP dispersion solution on their surfaces. Figure 1b shows the band structure of the PSCs, where some of the energy levels are derived from the UPS spectra (Fig. S3) and others are taken from the literature^37,38^. It is notable that the VBM of BP (−5.32 eV) matches very well with those of MAPbI_3_ perovskite (−5.50 eV) and spiro-OMeTAD (−5.15 eV). The cascade band structure of the multilayer device is favourable for the separation of electrons and holes and can reduce the carrier recombination rate at the interface between perovskite and hole transport materials (HTMs).

Figure 1c shows the representative current density–voltage (*J*–*V*) curves of PSCs without (control device) and with BP flakes modified on the perovskite surfaces. The amount of BP flakes on the surfaces was controlled by coating the solution for different times. The control devices exhibit only a moderate PCE (17.97%), while a clearly improved efficiency (19.85%) is obtained when the BP solution is coated one time. The PCE further improves to 20.32% when BP is spin-coated two times. However, the device performance cannot be further improved when BP is coated three times. The corresponding photovoltaic parameters for champion devices and the average values for 30 devices are listed in Table S1.

Figure 1d shows the external quantum efficiency (EQE) spectra of the above PSCs. In comparison with the control devices, a remarkable increase in EQE can be observed on the BP-modified devices over the whole wavelength range (from 300 to 800 nm), which explains the enhanced *J*_sc_ in the *J–V* characteristics. The integrated current density based on the highest EQE spectrum (two coats of BP) is 22.5 mA cm^−2^ (Fig. S5a), which is very close to the short-circuit current density presented in the *J–V* curve of the device.

Figure 1e shows the histogram statistics of PCEs for 30 devices fabricated with BP modification (two coats) and 30 control devices. The average PCE of the PSCs is improved by the BP flakes from 16.94 to 19.54% with a relative enhancement of 15.3%. By applying a voltage bias at the maximum power point (0.935 V) to the champion device, a stabilized photocurrent of 21.30 mA cm^−2^ and an efficiency of ~19.92% are obtained (Fig. S5b), which is equal to the average value of the PCEs obtained from the reverse (20.32%) and forward (19.53%) scans of the *J–V* curves. We also notice that the hysteresis of the *J*–*V* curves is greatly reduced due to the incorporation of BP flakes (Fig. S5c and Table S2). Control experiments indicate that the solvent IPA has no obvious effect on the performance of PSCs (Fig. S5d).

### Effect of BP flakes on perovskite grain boundaries

**Fig. 2:**
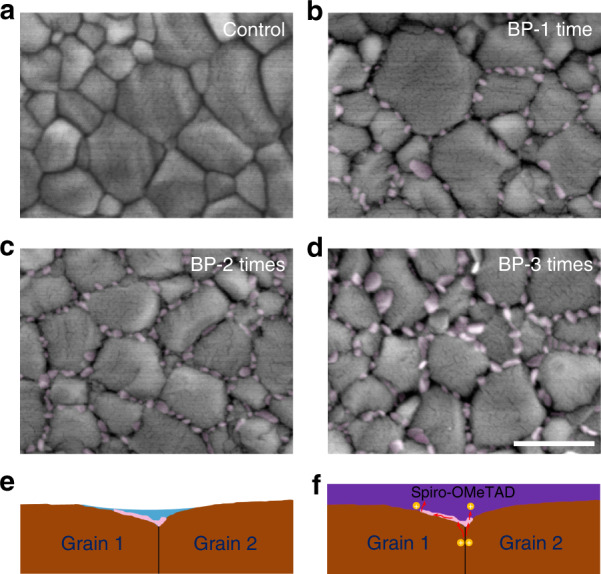
Morphology of the BP-modified MAPbI_3_ perovskite films. **a**–**d** SEM images of the perovskite films without and with 1–3 coats of BP flakes. The scale bar is 500 nm. **e** Schematic diagram of the grain boundary (GB) area when the BP dispersion in IPA was spin coated on the perovskite films. **f** Schematic diagram of the GB area after the deposition of BP and spiro-OMeTAD. Holes can transfer from the GB to the BP flake and then transport along the flake to the hole transport material spiro-OMeTAD

To shed light on the effect of BP flakes, the morphology of MAPbI_3_ perovskite films was characterized with scanning electron microscopy (SEM), as shown in Fig. 2. The SEM images demonstrate that the perovskite films have an average grain size of ~300 nm without pinholes. It is notable that the coverages of BP flakes on the film surfaces are only 4%, 8 and 10% for 1, 2 and 3 coats of the BP solution, respectively. Although the coverages are rather low, almost all the BP flakes are located on the GBs of the perovskite films. Notably, the space charge limited current (SCLC) characterizations of hole-only and electron-only devices (Fig. S6) confirm that the introduction of BP flakes at perovskite GBs has little effect on the electron and hole mobilities of the perovskite layer. Therefore, we believe the effect of BP flakes on PSCs is mainly due to the modification of GBs. The existence of BP flakes on the perovskite surface is also confirmed by energy-dispersive X-ray spectroscopy (EDX) mapping of phosphorene (Fig. S7). Notably, BP flakes on the GBs cannot passivate defects because they are physically absorbed on the edge of GBs, and no chemical bond can be formed with the perovskite layer, which is confirmed by Fourier transform infrared (FTIR) spectroscopy measurements (see Fig. S8).

To determine why the BP flakes are coated on the GBs only, we measured the contact angle of IPA on perovskite films with different crystallinities (Fig. S9). The average contact angles are 8°, 16° and 27° for the amorphous, polycrystalline and single-crystal perovskite surfaces, respectively, indicating that the contact angle of IPA decreases with the decreasing crystallinity of perovskite films. The surface energy of IPA on a perovskite film is related to the contact angle *θ* provided by Young’s equation^39^:

1 $$\cos \theta = \frac{{\gamma _{SV} - \gamma _{SL}}}{{\gamma _{LV}}}$$

where *γ*_*SV*_, *γ*_*SL*_ and *γ*_*LV*_ are the interfacial tensions of the solid–vapor, solid–liquid, and liquid–vapor interfaces, respectively, and *γ*_*SV*_ − *γ*_*SL*_ is the adhesion tension. Therefore, the adhesion tension increases with decreasing crystallinity. On the other hand, GB grooves can be formed during the crystallization process due to the diffusion of ions away from the boundaries^40^. Therefore, during the spin-coating process, the BP solution tends to move to GB regions, as shown in Fig. 2e, resulting in BP flakes mainly depositing on GBs after the evaporation of the IPA solvent.

Based on the above results, we present a schematic diagram in Fig. 2f to illustrate the mechanism for the performance enhancement induced by BP flakes in a PSC. Under light illumination, photo-generated holes tend to move to the GB and generate hole currents across BP flakes. Because BP has a much higher hole mobility (~1000 cm^2^V^−1^s^−1^) than spiro-OMeTAD (~10^−4^ cm^2^V^−1^s^−1^), BP flakes can conduct hole currents from GBs more efficiently. This model is also based on the assumption that the hole current density along a GB is higher than that in the adjacent grains due to band bending when near the GB (Fig. S10). To confirm this effect, we characterized our perovskite films on TiO_2_ by using c-AFM and find that the photo-induced hole current density at a GB is normally higher than that on adjacent grain surfaces, which is consistent with the results in the literature^41^. Moreover, the band banding that occurs close to a GB can enhance electron-hole separation due to the built-in electric field in this region. Therefore, GBs act as hole channels in perovskite active layers, and BP flakes can enhance hole transfer from GBs in the devices to improve device performance.

In addition, we are keen to know whether BP flakes can conduct electrons in PSCs to improve device performance. Hence, PSCs with an inverted structure of glass/ITO/poly[bis(4-phenyl)(2,4,6-trimethylphenyl)amine] (PTAA)/MAPbI_3_/BP/phenyl-C61-butyric acid methyl ester (PCBM)/bathocuproine (BCP)/Ag were prepared^42^. BP flakes were modified on the perovskite surfaces under the same conditions as the above experiments before coating the PCBM electron transport layers (ETLs). However, the performance of the inverted PSCs is not enhanced by the addition of BP flakes (Fig. S11), which can be attributed to the following two reasons. First, GBs in perovskite films can only conduct holes; thus, the modification of BP flakes on GBs beneath the ETL cannot facilitate the transfer of electrons in these devices. Second, the band structure of BP flakes is not favourable for electron transfer in these devices since the CBM level of BP is ~0.15 eV higher than that of the perovskite layer.

**Fig. 3:**
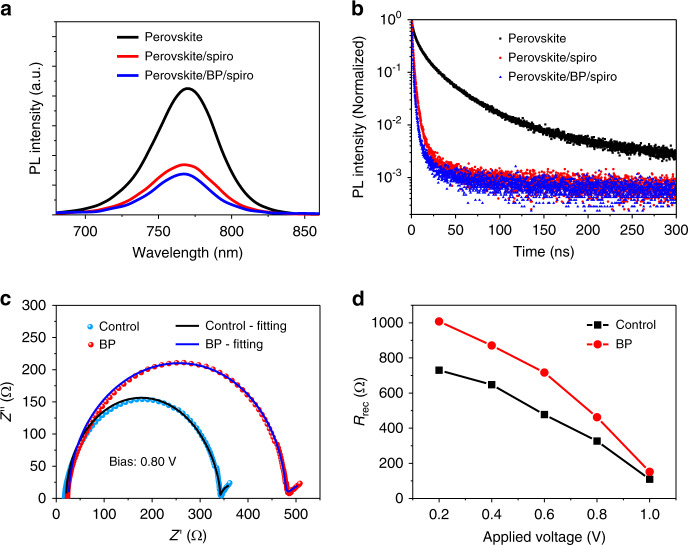
Optoelectronic characteristics of the MAPbI_3_ perovskite films and devices. **a**, **b** Steady state (**a**) and time-resolved (**c**) PL spectra of three films, including the perovskite, perovskite/spiro and perovskite/BP/spiro films; the films were deposited on quartz substrates. **c** Impedance spectra of the PSCs without (control) and with BP modification (2 coats) measured at a bias voltage of 0.80 V under light illumination of 100 mW cm^−2^. **d** Recombination resistance of PSCs derived from impedance spectra at different bias voltages under light illumination of 100 mW cm^−2^

To better understand the effect of BP flakes on MAPbI_3_ perovskite films, we characterized the steady-state and time-resolved photoluminescence (PL) behaviour of the following three samples: perovskite, perovskite/spiro-OMeTAD and perovskite/BP/spiro-OMeTAD films. As shown in Fig. 3a, the perovskite film without any modification exhibits the highest PL peak. The introduction of a spiro-OMeTAD layer on top of perovskite greatly reduces the PL intensity, which is further quenched by the presence of a BP layer, indicating more efficient hole transfer in the perovskite/BP/spiro-OMeTAD film. The time-resolved PL decay curves presented in Fig. 3b show a similar effect. The decay curves are fitted with a biexponential model: ${\mathrm{y}}\left( t \right) = y_0 + A_1e^{ - t/\tau _1} + A_1e^{ - t/\tau _2}$ (Fig. S12). The derived PL lifetime constants (*τ*_1_ and *τ*_2_) of the bare perovskite are 3.4 ns and 25.3 ns, which decrease to 1.4 ns and 9.2 ns by spiro-OMeTAD and further shorten to 0.8 ns and 2.9 ns after the BP modification. The improved PL quenching yield of the BP-modified devices indicates an enhanced hole transfer rate from perovskite to spiro-OMeTAD via the BP flakes on GBs. These results are consistent with the improved efficiency of BP-modified PSCs.

To further investigate the charge recombination behaviour of PSCs, we performed electrochemical impedance spectroscopy (EIS) measurements on the devices under light illumination of 100 mW cm^−2^. Figure 3c shows the EIS spectra of the devices at a bias voltage of 0.80 V. Two distinct regions can be observed in the Nyquist plots, including a high-frequency region correlated with the charge recombination process (large half circle) and a low-frequency region corresponding to the slow dielectric and ionic relaxation in perovskite films (part of the small circle)^43^. Figure 3d shows the recombination resistances (*R*_rec_) of the two PSCs at different bias voltages, which are derived from the Nyquist plots (Fig. S13). Both devices show a decreasing *R*_rec_ with an increasing bias voltage due to increased carrier densities^43,44^. A high *R*_rec_ value and long carrier lifetime can be observed in the BP-modified solar cell at any bias voltage, indicating a slow recombination rate being induced by BP flakes; thus, the improved hole extraction rate from perovskite to spiro-OMeTAD can be attributed to the BP flakes.

The stability of BP-modified PSCs was investigated in air. The statistical data of the PCEs of devices are presented in Fig. S14. The BP-modified PSCs and control devices can maintain ~92 and 76% of their initial average PCE after 1000 h, indicating that the devices with BP flakes are more stable than the control devices. This improved stability of the device can be attributed to the BP flakes that partially block the diffusion of outside ions, oxygen and moisture to the GBs^45–47^. On the other hand, Ahn et al. reported that the GBs in perovskite films are unstable in the simultaneous presence of charge and moisture^48^. BP flakes can efficiently conduct holes and decrease the density of holes accumulated in GBs, which can be another reason for the improved stability of BP-modified devices.

### Grain-size effect of perovskite films with the BP modification

**Fig. 4:**
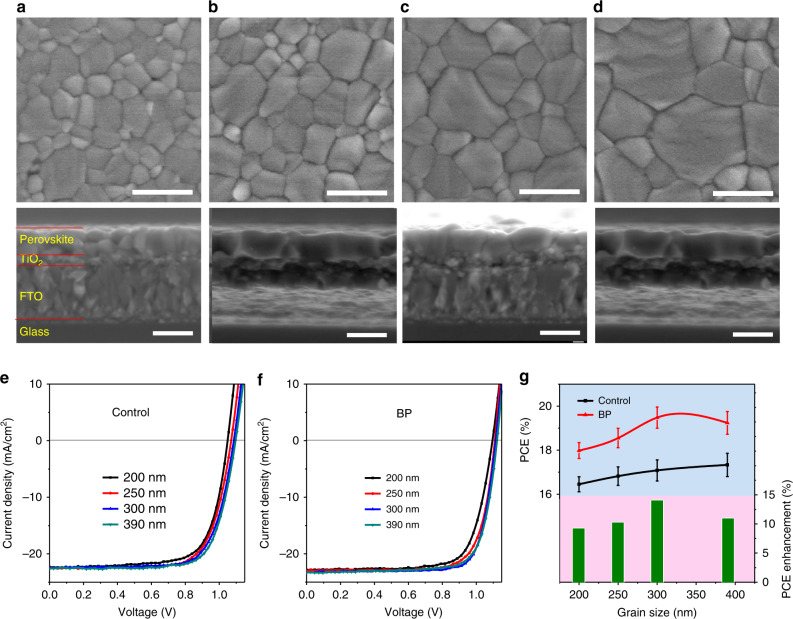
Grain-size effect of MAPbI_3_ perovskite films. **a**–**d** Plan view and corresponding cross-sectional view of the scanning electron microscopy (SEM) images of perovskite films with different grain sizes on FTO/TiO_2_ substrates. The length of all the scale bars is 500 nm. The average grain sizes of the perovskite films in **a**–**d** are 200, 250, 300 and 390 nm, respectively. **e**, **f** *J*–*V* curves of the PSCs without (control) and with BP (2 coats) deposited on perovskite films. **g** Average PCEs and relative PCE enhancement (after the BP modification) of PSCs with different perovskite grain sizes. The black curve (control) and red curve (BP) correspond to the PCEs of devices without and with the BP modification, respectively, while the green columns represent the relative PCE enhancement after the BP modification

To demonstrate the effect of GBs in PSCs, we prepared MAPbI_3_ perovskite films with different grain sizes by controlling the thermal annealing periods. As shown in Fig. 4, perovskite films with average grain sizes of 200, 250, 300 and 390 nm were prepared after thermal annealing at 100 °C for 10, 30, 60 and 120 min, respectively. For the devices without the BP flake modification, the device efficiency increases with an increasing grain size (Table S3 and Table S4), which is consistent with the results in the literature^42,49^. After the BP flake modification under the same conditions, the PCEs of the devices dramatically improve. When the average grain size is ~300 nm, the devices show the highest PCE and the maximum relative enhancement. This result indicates that a suitable number of GBs is favourable for the performance of PSCs because the photocurrents in the devices can be conducted out along the GBs (Fig. S10). Notably, devices with smaller grain sizes (200 and 250 nm) show lower PCE enhancements when the GBs are modified with BP flakes. We can find the reason from the cross-sectional SEM images of the perovskite films shown in Fig. 4. When the grain size is smaller than 300 nm, the perovskite grains cannot penetrate the films from the bottom to the top. In this case, the GBs in the middle will prohibit electron transport in the devices, although they continue to be hole conductors (Fig. S10f)^50^.

### Modification of other 2D material flakes

Solutions of other 2D materials, including MoS_2_, GO and BP quantum dots (BP-QDs), were prepared and modified on the perovskite layers of PSCs with the same procedure^51–53^. As expected, almost all of the MoS_2_ and GO flakes are located on the perovskite GBs (Fig. S15). The average sizes of the MoS_2_ and GO flakes are estimated to be ~40 nm. However, BP-QDs are too small to be observed with SEM. The average size of BP-QDs observed with TEM is less than 5 nm (Fig. S16). It is reasonable to expect that BP-QDs are located on the GBs of perovskite films as well. The *J*–*V* curves and photovoltaic parameters of the PSCs modified with MoS_2_, GO and BP-QDs under optimum coating conditions were characterized (Fig. S17 and Table S5). The photovoltaic performances of the devices are improved by all the 2D materials, and the PCE enhancements are presented in Fig. 1f. Notably, BP flakes can induce better photovoltaic performance than other 2D materials, which can be attributed to BP having the highest carrier mobility. Moreover, BP-QDs can only conduct holes at a distance of several nanometres, and thus, their effect is inferior to that of BP flakes.

### Device simulation for the effect of 2D flakes

**Fig. 5:**
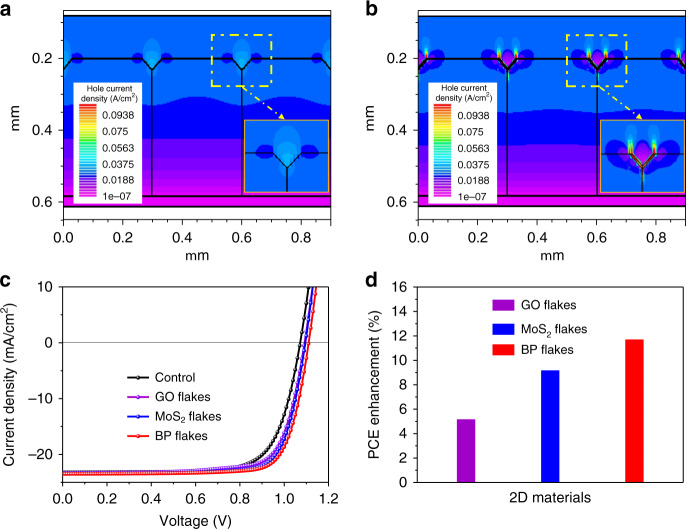
Device simulation results of the MAPbI_3_ PSCs. **a**, **b** Hole current density distribution of the simulated PSCs without (**a**) and with (**b**) the BP modification. **c** Simulated *J–V* curves of the PSCs modified with different 2D materials. **d** Relative PCE enhancement of the PSCs after modifications with different 2D materials

To better understand the performance enhancement of MAPbI_3_ PSCs induced by 2D flakes modified on GBs, we performed a device simulation by using the commercial software Silvaco. A device with a perovskite grain size of 300 nm was designed for simulation (Fig. S18, Table S6 and Table S7). The GB grooves were assumed to be 30 nm deep, which is consistent with our AFM measurements. To simulate the effect of BP in the devices, a BP layer was added on the surfaces of these grooves. As shown in Fig. 5, the *J–V* curves of the devices are simulated before and after coating the BP layer under standard illumination conditions (AM 1.5 G, power density: 100 mW cm^−2^). It is interesting to find that the simulated performance is very similar to our experimental results (Table S8). After the BP modification, the open-circuit voltage and the fill factor of the device are substantially improved. We can clearly find high hole currents conducted out by the BP flakes from the GBs in Fig. 5b. Here, the hole mobility of BP is ~1000 cm^2^V^−1^s^−1^, which is 7 orders of magnitude higher than that of spiro-OMeTAD^29^. When we replaced BP with MoS_2_, which has a hole mobility of ~70 cm^2^V^−1^s^−1^ ^54–56^, the PCE enhancement is lower than that provided by BP. We also simulated the effect of GO flakes (hole mobility is assumed to be ~0.015 cm^2^V^−1^s^−1^) on GBs, and a performance enhancement is observed due to the hole conduction of GO^57^. Our simulation results demonstrate that the performance enhancement induced by 2D flakes increases with an increase in the hole mobilities of 2D flakes, which is consistent with our experimental results presented in Fig. 1f. Notably, all conventional HTMs, including Spiro-OMeTAD, PTAA, poly(3,4-ethylene dioxythiophene):poly(styrene sulfonate) (PEDOT:PSS), poly(3-hexylthiophene) (P3HT), NiO_x_ and CuSCN, which are normally used in PSCs, have hole mobilities of less than 0.01 cm^2^V^−1^s^−1^ (Table S9). In this case, the hole currents from GBs cannot be efficiently conducted out by the HTMs; thus, the advantage of GBs that can form vertical hole channels in PSCs cannot be observed.

### Mixed perovskite solar cells modified with 2D flakes

We then tested the effect of 2D flakes in (CsPbI_3_)_0.05_(FAPbI_3_)_0.95_(MAPbBr_3_)_0.05_ mixed PSCs with high efficiency^58^. The mixed PSCs have the normal device structure shown in Fig. 1a. Similarly, different 2D materials dispersed in IPA were spin coated on the perovskite surfaces. Notably, most of the 2D flakes are located on the GBs of perovskite films (Fig. S19a, b). The 2D material interlayer can also form a cascade band structure with the (CsPbI_3_)_0.05_(FAPbI_3_)_0.95_(MAPbBr_3_)_0.05_ mixed perovskite and spiro-OMeTAD films (Fig. S3h), which will be favourable for charge separation and transport. Similar to the MAPbI_3_ devices, we find that 2 coats of 2D flakes on the mixed perovskite leads to the best effect (see Fig. S20).

**Fig. 6:**
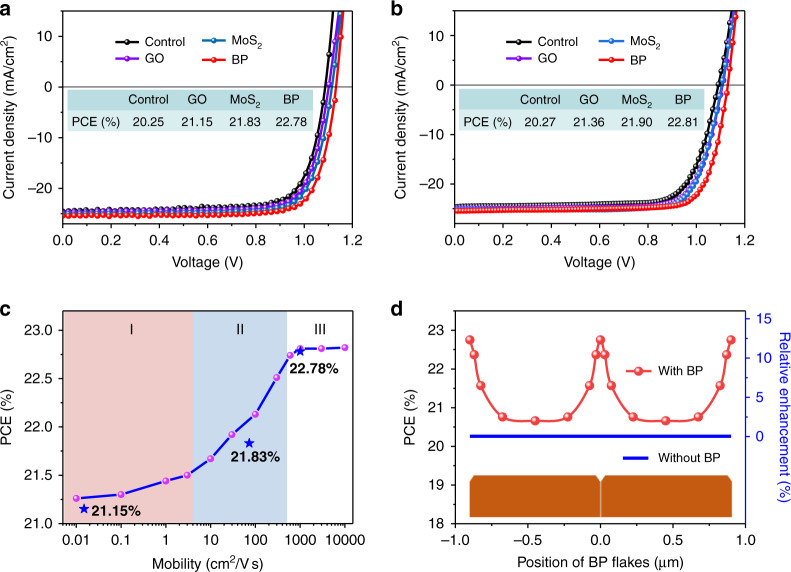
Photovoltaic performances of the mixed PSCs. **a**, **b** Experimental (**a**) and simulated (**b**) *J–V* curves of the mixed PSCs modified with different 2D flakes on perovskite GBs. **c** Simulated device efficiencies of the mixed PSCs with varying mobilities of 2D flakes. The three regions (I, II and III) correspond to the carrier mobilities of the three different 2D flakes (GO, MoS_2_ and BP), and the star symbols represent the experimental efficiencies of the PSCs modified with the three different 2D flakes in 6a. **d** Simulated device PCEs and efficiency enhancement for BP flakes located at different positions on the perovskite grains. The length of a single BP flake is ~42 nm in all simulations

As shown in Fig. 6a, the pristine mixed PSCs have the best PCE of 20.25%, *V*_oc_ of 1.09 V, *J*_sc_ of 24.56 mA cm^−2^ and FF of 75.6%, while the BP-modified champion device exhibits a high PCE of 22.78%, *V*_oc_ of 1.13 V, *J*_sc_ of 25.42 mA cm^−2^ and FF of 79.3%, with negligible *J–V* hysteresis (ΔPCE = PCE_reverse_ – PCE_forward_ = 0.05%, see Fig. S19c). A stabilized efficiency of 22.5% is achieved when a bias voltage of 0.96 V is applied to the champion device (Fig. S19d). Similar to the PSCs based on MAPbI_3_, the stability of the mixed PSCs was also improved by BP flakes (Fig. S21). For the light stability test, the BP-modified device can maintain ~91% of its initial PCE after light soaking tests for over 400 h, while the control device can only retain ~65% of its original efficiency (Fig. S21c). It is reasonable to find that the performance enhancements induced by MoS_2_ and GO flakes are also obvious, and the photovoltaic parameters are shown in Table S10.

As shown in Fig. 6b, mixed PSCs with different 2D flakes are simulated with Silvaco, and the photovoltaic parameters enhanced by the 2D flakes fit very well with the experimental results. Considering that the carrier mobility of a 2D flake varies with its layer number and size, we assumed that the carrier mobilities of 2D flakes changed from 0.01 to 10^4^ cm^2^V^−1^s^−1^ in the simulation and obtained the relationship between the device efficiency and hole mobility (Fig. S22a and Table S11). As shown in Fig. 6c, regions I, II and III correspond to the carrier mobilities of the three different 2D flakes, namely, GO, MoS_2_ and BP, respectively. The device efficiency increases with an increasing carrier mobility, while the enhancement is saturated when the mobility is over 10^3^ cm^2^V^−1^s^−1^. Therefore, BP should be the optimum 2D material for the conduction of carriers from the GBs in PSCs.

Notably, the performance enhancement induced by BP flakes cannot be simply attributed to the cascade band structure of the perovskite/BP/spiro-OMeTAD interface because the coverage of 2D flakes on the surface of a perovskite film is less than 10%. To better understand the underlying mechanism, we further simulated the effect of the location of BP flakes on the device performance. Schematic diagrams of the device structures (without BP and with BP flakes at different positions) and corresponding hole current density distribution are shown in Fig. S23. High hole current densities can be found at positions near BP flakes in the simulated mixed PSCs due to enhanced hole conduction at the perovskite/BP/spiro-OMeTAD interface. Compared with control devices (without BP), the highest relative PCE enhancement (~12.5%) can be obtained when BP flakes are located exactly on GBs (Fig. 6d, Fig. S22b and Table S12), which is consistent with our experimental results shown above. In contrast, only an ~2% relative PCE enhancement can be achieved when the BP flakes are located at the centre of grain surfaces. Therefore, the synergistic effect of BP flakes on GBs in a PSC plays an essential role in performance enhancement.

## Discussion

We developed a convenient and cost-effective approach to modify the GBs of perovskite films by using high-mobility 2D flakes. Although the coverage of 2D flakes on the perovskite films was only several percent, most of the flakes were located on the perovskite GBs. Due to the high carrier mobilities of 2D materials, especially BP, the hole transfer from GBs was dramatically enhanced in PSCs, resulting in substantial improvements in the efficiency and stability of the devices. Our results also indicated that the GBs in PSCs were not detrimental to the device performance if the accumulated holes in the GBs could be efficiently conducted out. Under certain conditions, GBs could even be favourable for the photovoltaic performance of PSCs due to the built-in electric fields around them, which could facilitate photocarrier separation and transfer in devices. Therefore, the perovskite GBs were electrically benign, which was consistent with previously reported theoretical calculations^59–61^. Notably, we observed the synergistic effect of 2D flakes on GBs in PSCs for the first time. Both the carrier mobility and the location of the 2D flakes on the perovskite surface were essential to the performance enhancement. This work provides a guideline for modifying perovskite layers with novel high-mobility 2D materials to improve the photovoltaic performance and stability of PSCs.

## Materials and methods

### Materials

Methylammonium iodide (MAI), formamidinium iodide (FAI), methylammonium bromide (MABr) and TiO_2_ paste (30 NR-D) were ordered from Greatcell Solar, Ltd, Australia. PbI_2_ (99.999%) and dimethylformamide (DMF) were purchased from Alfa Aesar, UK. CsI (99.999%), dimethylsulfoxide (DMSO), chlorobenzene, titanium isopropoxide, 4-tert-butylpyridine (tBP) and bis(trifluoromethylsulfonyl)imide lithium salt (Li-TFSI) were purchased from Sigma–Aldrich, Ltd, USA. Methylammonium chloride (MACl) and PbBr_2_ (99.98%) were purchased from Xi’an Polymer Light Technology Corp, China. Spiro-OMeTAD was purchased from Taiwan Lumtec, Ltd, China.

### Preparation of 2D materials

Commercialized bulk BP crystals were used for the preparation of BP thin flakes. Ten milligrams of the BP crystals were first ground with a mortar in a glovebox, dispersed in 5 ml anhydrous IPA, and then ultrasonicated for ~20 h with an ultrasonic disrupter (BioSafer 650-92, output power ~400 W) at low temperature (maintained by an ice-water bath). The solution was then centrifuged at 3000 rpm for 40 min, and the upper dispersion was collected and re-centrifuged at 6000 rpm for 40 min. The BP thin flakes in the upper dispersion solution were carefully collected for tests and solar cell applications. MoS_2_, GO flakes and BP-QDs were prepared using a similar procedure.^51–53^ The concentration of 2D flakes was approximately 0.1 mg/ml (measured by filtration and weighing).

### Device fabrication

PSCs with a normal structure in this work have a configuration of glass/FTO/TiO_2_/perovskite/spiro-OMeTAD/Au. The FTO substrates (14 Ω/sq were cleaned with soap water, distilled water, acetone and IPA and treated with O_2_ plasma for 5 min. A thin c-TiO_2_ film (~30 nm) was then deposited on the FTO glass by spin coating at 4000 rpm from a solution of 0.15 M titanium isopropoxide in ethanol (with the addition of 1.5 mM HCl from 37 wt% hydrochloric acid) and was sintered at 500 °C for 30 min in an oven. After cooling to room temperature, a mp-TiO_2_ layer (~150 nm) was spin coated on cp-TiO_2_ at 4000 rpm by using TiO_2_ paste (Dyesol 30 NR-D, 30-nm nanoparticles) diluted in tert-butanol (weight ratio 1:7) and sintered again at 500 °C for 30 min. After cooling down again, the substrates were immersed in a 40 mM clear aqueous solution of TiCl_4_ for 30 min at 70 °C, washed with distilled water and IPA, blow-dried with air flow and sintered again at 500 °C for 30 min. Upon cooling to 150 °C, the substrates were immediately transferred into a glovebox for perovskite film deposition. The perovskite precursor was prepared by dissolving 0.5763 g of PbI_2_ and 0.1986 g of MAI in 1 ml of mixed solvents of anhydrous DMF and DMSO (volume ratio, 8:1). After filtering, 60 μl of a CsPbI_3_ solution (1 M, dissolved in DMF and DMSO, 4:1) was added to the solution. The Cs-doped MAPbI_3_ precursor was spin coated on top of the TiO_2_ substrates at 4000 rpm for 30 s in a glovebox. After 10 s of the spin-coating process, approximately 150 μl of chlorobenzene was poured onto the spinning substrate. The substrates were then annealed on a hotplate at 65 °C for 2 min followed by 100 °C for 60 min in a glovebox. In the experiments of the grain-size effect, the annealing time at 100 °C was tuned from 10 min to 120 min. To prepare (CsPbI_3_)_0.05_(FAPbI_3_)_0.95_(MAPbBr_3_)_0.05_ mixed perovskite films, 1.013 g of FAPbI_3_ yellow powder^62^, 41 mg of MAPbBr_3_ single crystals^63^ and 38 mg of MACl were dissolved in mixed solvents of 0.89 ml of DMF and 0.11 ml of DMSO. After filtering, 80 μl of a CsPbI_3_ solution (1 M, dissolved in DMF and DMSO, 4:1) was added to obtain the final mixed perovskite solution. The mixed solution was then spin coated on the mp-TiO_2_ films at 1000 rpm for 5 s and 5000 rpm for 25 s. After 20 s of the spin-coating process, 0.5 ml ethyl ether was poured onto the spinning substrates. The perovskite precursor films were then annealed at 150 °C for 10 min. For the BP-modified devices, the freshly prepared BP dispersion was spin coated on perovskite films at 4000 rpm, followed by annealing at 70 °C for 5 min. This process may be repeated several times to coat BP flakes with different amounts. After the BP flake modification, a spiro-OMeTAD (75 mg ml^−1^ in chlorobenzene) solution was spin coated on top at 4000 rpm. The spiro-OMeTAD solution (1 ml) was doped with 28.5 μl of tBP and 17.5 μl of Li-TFSI (520 mg ml^−1^, dissolved in acetonitrile). Finally, a gold electrode (~100 nm) was deposited on top through a shadow mask by thermal evaporation. The active area (the overlapping area between the FTO and Au contacts) of an individual solar cell was 8 mm^2^. Each substrate was then encapsulated with epoxy and glass covers. The fabrication process of PSCs with other 2D materials was similar to that of the BP-modified devices. MoS_2_, GO or BP-QDs dispersions in IPA were spin coated on perovskite films before the deposition of spiro-OMeTAD and Au electrodes. In addition, hole-only devices (ITO/PTAA/MAPbI_3_/Au; ITO/PTAA/MAPbI_3_/BP/Au) and electron-only devices (ITO/SnO_2_/MAPbI_3_/PCBM/Ag; ITO/SnO_2_/MAPbI_3_/BP/PCBM/Ag) were prepared and tested for space charge limited current (SCLC) measurements.

### Material and film characterizations

AFM measurements were carried out with a Bruker Nanoscope 8 system (tapping mode). TEM observations were carried out by using a JEOL JSM 2100 F scanning transmission electron microscope operating at 200 kV. SEM images were obtained by using a field-emission scanning electron microscope (Hitachi S-4300). FTIR measurements were performed using a Bruker Fourier transform infrared spectroscopy system. UPS measurements were carried out with a VG ESCALAB 220i-XL ultrahigh vacuum surface analysis system equipped with a He-discharge lamp providing He-I photons at 21.22 eV. The base vacuum of the system was ~10^−10^ Torr, and a −5.0 V bias was applied during the measurements. The PL measurements were carried out by using an Edinburgh FLSP920 fluorescence spectrophotometer. A 636-nm laser was used as an excitation light source. The c-AFM measurements were performed by using an MFP-3D series instrument (Asylum Research) with light illumination from the side of the perovskite film. The used AFM tips were coated with Cr/Pt, and no bias voltage was applied during the test.

### Device characterization

The *J–V* curves of the PSCs were measured in ambient air with a Keithley 2400 source meter under light illumination of 100 mW cm^−2^ (Newport 91160 solar simulator, 300 W, equipped with an AM 1.5 G filter). The light source was calibrated frequently during the *J–V* tests by using a standard silicon reference cell (also calibrated) to ensure accurate light intensity. The *J–V* characteristics were determined by applying an external voltage while measuring the current response. For a standard sweeping cycle, the external applied bias changed from 1.2 to 0 V (reverse scan) and then returned to 1.2 V (forward scan) with a voltage scan rate of 30 mV s^−1^. No preconditioning (such as forward bias or light soaking) was applied before the measurements. EQE spectra were measured with a standard EQE test system from Newport, consisting of a Xenon lamp (Oriel 66902, 300 W), a monochromator (Newport 66902), a silicon detector (Oriel 76175_71580) and a dual-channel power meter (Newport 2931_C). EQE measurements were performed in DC mode (300–800 nm) at room temperature. Stable power output characteristics were carried out at the maximum power point of the device under standard light illumination of 100 mW cm^−2^, and the current response was recorded as a function of time by a Keithley 2400 source meter. Impedance measurements of the PSCs were carried out under light illumination of 100 mW cm^−2^ (white light) by using a Zahner Zennium 40630 electrochemical workstation. The oscillating voltage was 50 mV, and the applied DC bias voltages varied from 0 to 1.0 V. The frequency of the sinusoidal signal changed from 2 MHz to 1 Hz. For solar cell stability tests, all devices were encapsulated with epoxy/glass.

### Device simulation

Silvaco TCAD 2014 software was used to model and simulate PSCs with a structure of FTO/TiO_2_ (30 nm)/perovskite (380 nm)/spiro-OMeTAD (120 nm)/Au electrode. The device structure and two-dimensional mesh were constructed with the DevEdit module. The output structure file was imported by the ATLAS module followed by setting parameters for user-defined materials and declaring physical models to be calculated. The Luminous module integrated in the ATLAS framework was used to simulate light propagation and absorption. The *J–V* characteristics of the devices were measured under simulated solar irradiation (AM 1.5 G, 100 mW cm^−2^) with light illuminated from the FTO/TiO_2_ side. The grain boundaries were simulated by creating a narrow region with a high trap density between two adjacent perovskite grains. We assumed interfacial p-type doping in the GB regions (thickness is 1 nm) with a doping level of 10^15^ cm^−3^ at an energy level of 0.1 eV above the VBM. The thickness of each layer and the dimensions of the grain boundary region are presented in Table S7 and Fig. S23. The recombination of carriers in the body part and at the interfaces was considered by calculating SRH and Auger recombination. Finally, TonyPlot was used to visualize the results written in the log file output by ATLAS. All diagrams except the *J–V* curves were extracted at a bias voltage of zero. The material parameters used are listed in Table S6 and Table S7.

## Supplementary information

Supplementary Information for 2D Materials for Conducting Holes from Grain Boundaries in Perovskite Solar Cells

## Data Availability

The authors declare that the data supporting the findings of this study are available within the paper and its supplementary information files.
