# Supplementary material for: 2D materials for conducting holes from grain boundaries in perovskite solar cells
Source: Light Sci Appl. 2021 Mar 31;10:68. doi: 10.1038/s41377-021-00515-8 (PMC8012639; doi:10.1038/s41377-021-00515-8)
Supplement: Supplementary file 1 — Supplementary Information for 2D Materials for Conducting Holes from Grain Boundaries in Perovskite Solar Cells [file 41377_2021_515_MOESM1_ESM.docx]

Supplementary Information for

**2D Materials for Conducting Holes from Grain Boundaries in Perovskite Solar Cells**

Peng You,^1,2^ Guanqi Tang,^1^ Jiupeng Cao,^1^ Dong Shen,^3^ Tsz-Wai Ng,^3^ Zafer Hawash,^4^ Naixiang Wang,^1^ Chun-Ki Liu,^1^ Wei Lu,^5^ Qidong Tai,^1^ Yabing Qi,^4^ Chun-Sing Lee,^3^ Feng Yan^1^*

*^1^Department of Applied Physics, The Hong Kong Polytechnic University, Hung Hom, Kowloon, Hong Kong, China.*

*^2^College of New Materials and New Energies, Shenzhen Technology University, Shenzhen 518118, China.*

*^3^Center of Super-Diamond and Advanced Films (COSDAF), Department of Chemistry, City University of Hong Kong, Hong Kong, China.*

*^4^Energy Materials and Surface Sciences Unit (EMSSU), Okinawa Institute of Science and Technology Graduate University (OIST), 1919-1 Tancha, Onna-son, Okinawa 904-0495 Japan.*

*^5^University Research Facility in Materials Characterization and Device Fabrication, The Hong Kong Polytechnic University, Hung Hom, Kowloon, Hong Kong, China.*

*E-mail: [apafyan@polyu.edu.hk](mailto:apafyan@polyu.edu.hk)

**Content:**

1. **Characterization of 2D flakes (Fig. S1-S4)**
2. **Characterization of MAPbI_3_ films and PSCs (Fig. S5-S17)**
3. **Device Simulation of MAPbI_3_ PSCs (Fig. S18)**
4. **Characterization of mixed perovskite films and PSCs (Fig. S19-S21)**
5. **Device simulation of mixed PSCs (Fig. S22-S23)**
6. **Supplementary Tables**
7. **References**
8. **Characterization of 2D flakes:**


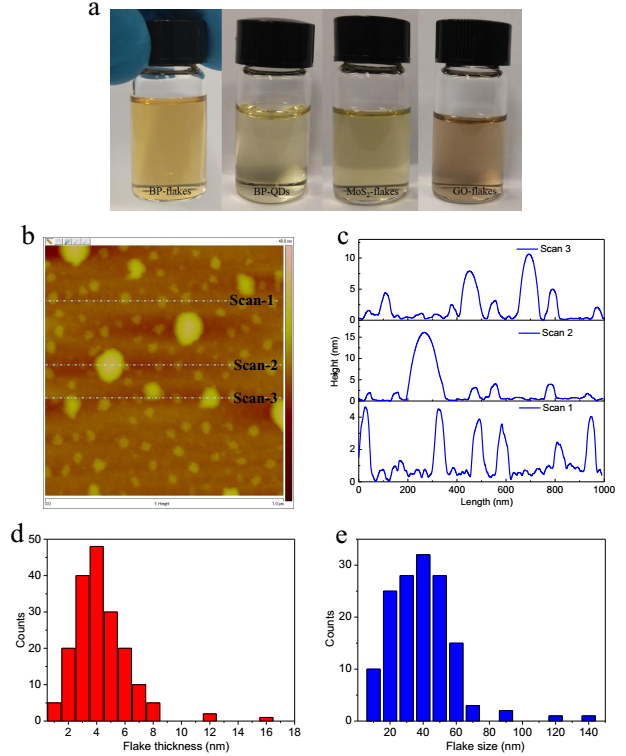


**Fig. S1 | Characterization of the size of BP flakes.**

**a**, Photos of dispersion of different 2D materials in anhydrous IPA. **b**, AFM image of BP flakes on Si/SiO_2_ substrate. **c**, The height profile obtained from figure a. **d**,**e**, The thickness and size distribution graphs of the BP flakes measured from figure a.

Note: Samples for AFM measurements were prepared by spin coating BP dispersion on clean Si/SiO_2_ (300-nm SiO_2_) substrates at 4000 rpm for 30 s in the glovebox and annealed at 70ºC for 5 min


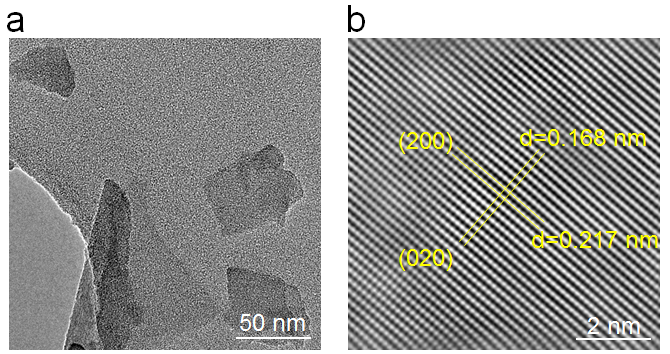


**Fig. S2 | TEM characterization of BP flakes.**

**a**, Bright field TEM image of the BP flakes. **b**, High resolution TEM image of a BP flake (after Fourier filtering).


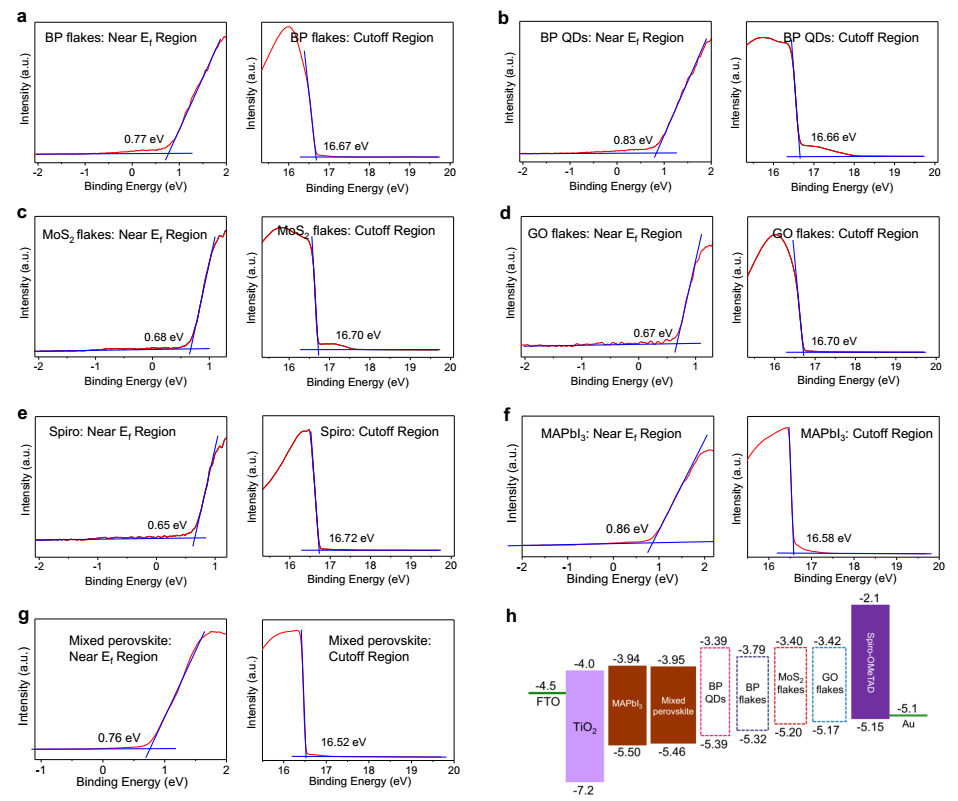


**Fig. S3 | UPS characterization of 2D materials and perovskites.**

**a-g**, Ultraviolet photoelectron spectroscopy (UPS) spectra of different 2D materials, spiro-OMeTAD and perovskite films. **h**, The energy level band diagram of the PCSs with different 2D materials, where some of the energy levels are taken from literature^1-3^. The films for UPS measurements were prepared by spin coating 2D flake dispersion on ITO substrates in a glovebox and annealed at 70 ºC for 5 min.


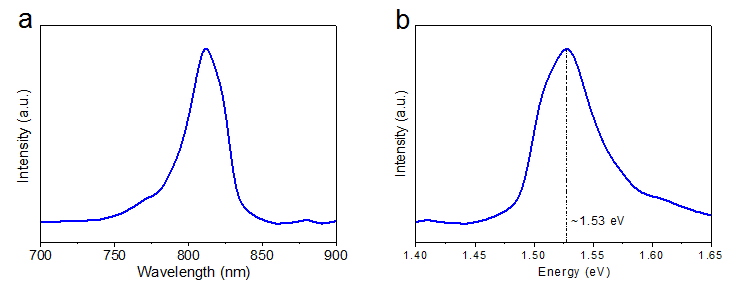


**Fig. S4 | PL spectra of BP flakes.**

**a**, The original Photoluminescence (PL) spectrum of BP film on Si/SiO_2_ substrate (300-nm SiO_2_). **b**, The converted PL spectrum, showing the relationship between PL intensity and energy.

1. **Characterization of MAPbI_3_ films and PSCs:**


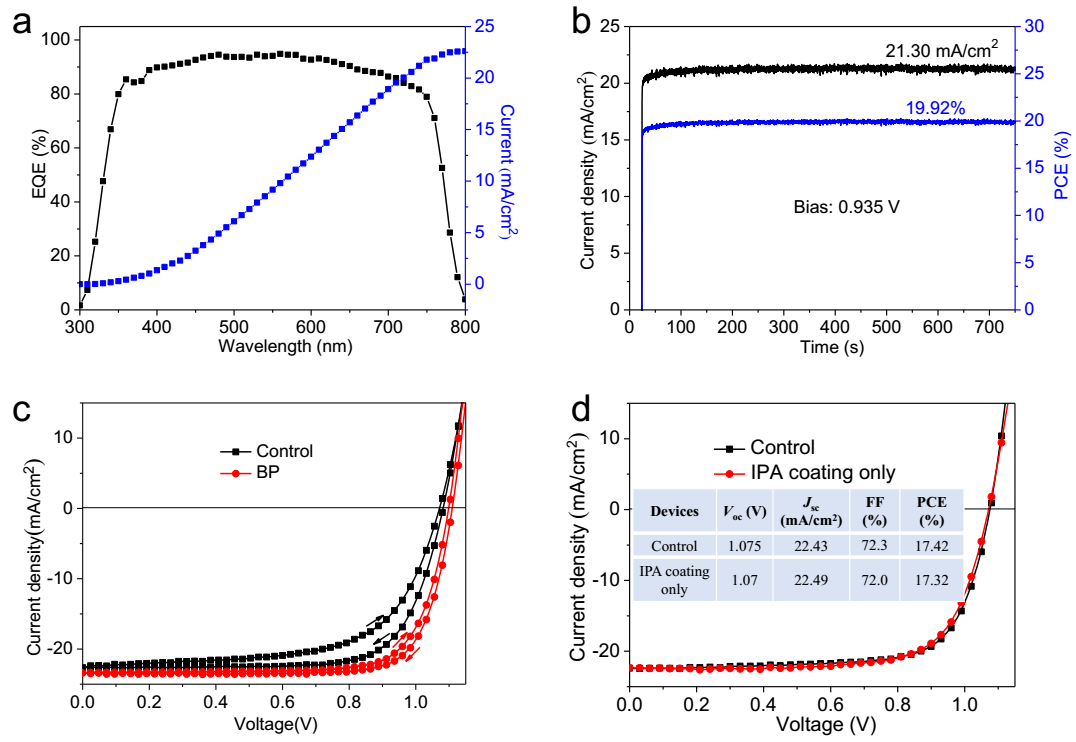


**Fig. S5 | Photovoltaic performance of MAPbI_3_ PSCs.**

**a**, EQE curve and the integrated photocurrent from one of the best BP-modified MAPbI_3_ PSCs. **b,** Steady-state photocurrent and stabilized output efficiency of the best MAPbI_3_ PSC measured at a bias voltage near the maximum power point (0.935 V).

**c**, *J-V* hysteresis of MAPbI_3_ PSCs without (control) and with BP deposition (2 times coating) on perovskite surface. **d**, *J-V* curves and photovoltaic parameters of MAPbI_3_ PSCs without (control) and with IPA coating on perovskite surface.


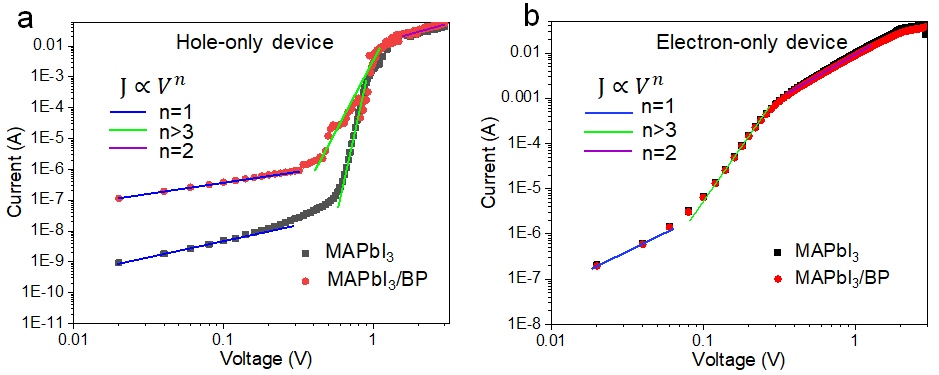


**Fig. S6 |** Space-charge-limited currents (SCLC) measurements of hole-only and electron-only devices. The hole-only (ITO/PTAA/MAPbI_3_/Au; ITO/PTAA/MAPbI_3_/BP/Au) and electron-only (ITO/SnO_2_/MAPbI_3_/PCBM/Ag; ITO/SnO_2_/MAPbI_3_/BP/PCBM/Ag) devices are prepared respectively. The above current versus voltage curves measured by SCLC method consist of three regimes: (1) a linear ohmic regime (n=1); (2) a trap-filled regime (n>3); (3) a trap free SCLC regime to demine the charge mobility at high bias (n=2). The SCLC mobility can be calculated using equation ${J=\frac{9}{8}\mu\varepsilon}_{0}\varepsilon_{r}\frac{V^{2}}{L^{3}}$ , where *μ* is the carrier mobility, *V* is the applied voltage, *ε*_0_ is the free space permittivity, *ε*_r_ is the dielectric constant of the perovskite, L is the thickness of the perovskite layer. For control devices without BP, the calculated electron and hole mobilities of perovskite films are 14.1*10^-4^ cm^2^ V^-1^ s^-1^ and 9.6*10^-4^ cm^2^ V^-1^ s^-1^, respectively. And for devices modified with BP flakes, the calculated electron and hole mobilities of perovskite films are 12.3*10^-4^ cm^2^ V^-1^ s^-1^ and 10.0*10^-4^ cm^2^ V^-1^ s^-1^, respectively. Therefore, the electron and hole mobilities of perovskite films are comparable, and the introduction of BP flakes on the perovskite grain boundaries has little effect on the carrier mobility of perovskite films.


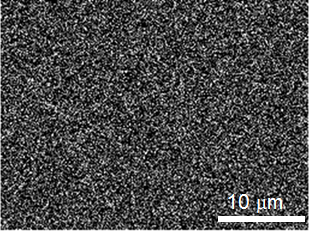


**Fig. S7 |** SEM EDX mapping of phosphorene element for BP-modified MAPbI_3_ perovskite films.


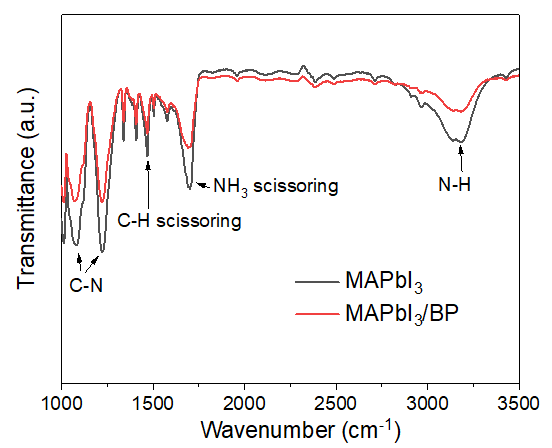


**Fig. S8 |** Fourier Transform Infrared Spectroscopy (FTIR) spectra of MAPbI_3_ perovskite films before and after modification of BP flakes.


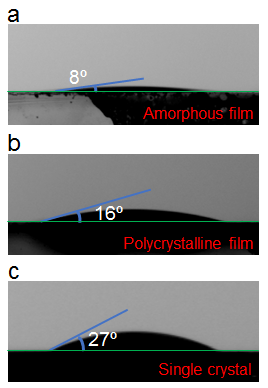


**Fig. S9 | Contact angle of IPA on different perovskite surfaces.**

**a**-**c**, Contact angle of IPA on (**a**) amorphous, (**b**) polycrystalline and (**c**) single-crystal MAPbI_3_ perovskite surfaces.

**
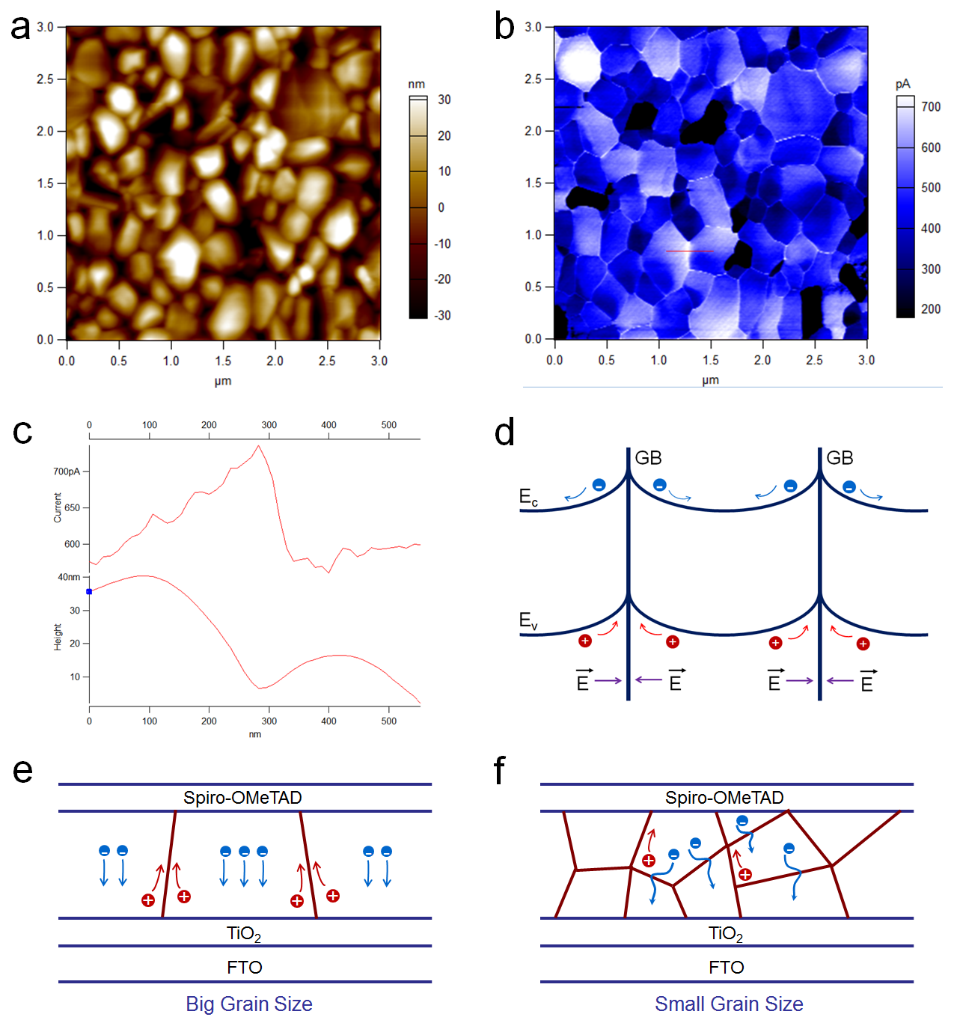
**

**Fig. S10 | C-AFM characterization on MAPbI_3_ films.**

The conductive atomic force microscopy (c-AFM) measurements were performed on a glass/FTO/TiO_2_/MAPbI_3_ surface. **a**,**b**, The topographic image and current image taken under illumination without applied bias voltage. **c**, The current and height profile taken from the grain boundary area marked in figure b. **d**, The schematic band structure of the GB region, where build-in electric field is formed due to the p-type doping of the GBs. **e**,**f**, The schematic diagram of the charge transport processes in PSCs with small and big perovskite grain sizes. For PSCs with small perovskite grain sizes, there is a potential barrier when electrons transport through the GBs between two stacked perovskite grains, as shown in figure f, while for PSCs with big grain sizes, most of the perovskite grains are penetrating through the perovskite film (without grain stacking, as shown in figure e), which are beneficial for the charge transport from the perovskite material to the charge collection layers.


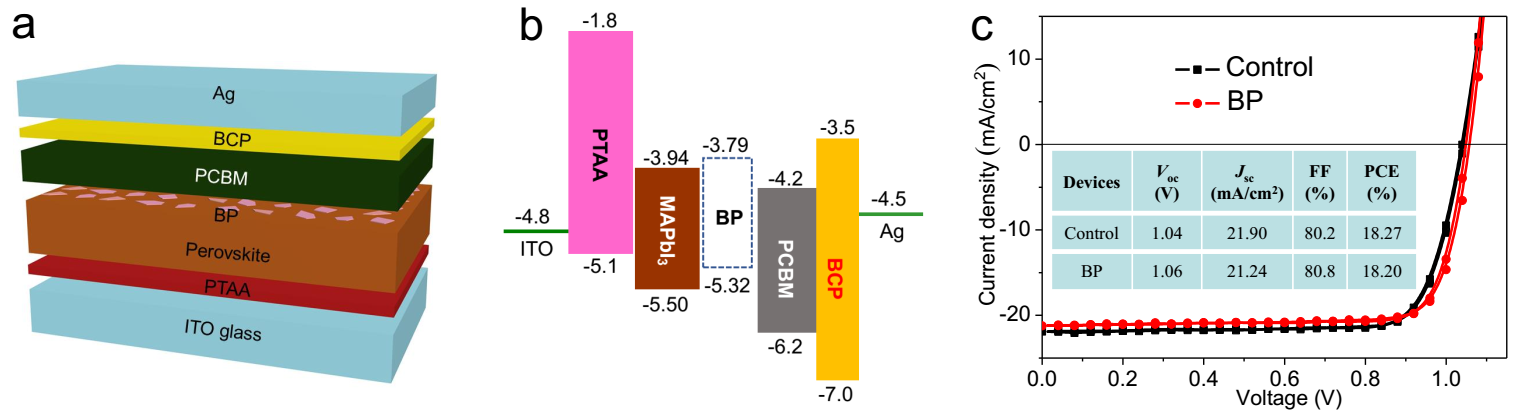


**Fig. S11 | Photovoltaic performance of inverted MAPbI_3_ PSCs.**

**a**,**b,** The device structure and the corresponding energy band diagram of the inverted-structure MAPbI_3_ PSCs modified with BP flakes. **c**, *J-V* curves and photovoltaic parameters of the inverted PSCs.


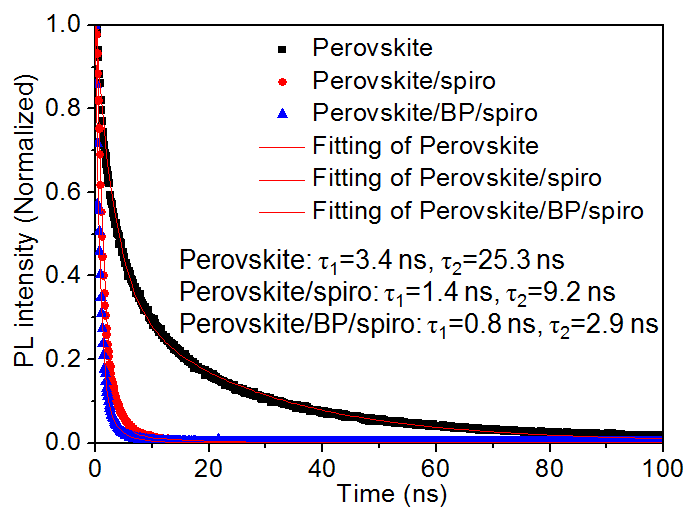


**Fig. S12 | Time-resolved PL characterization results of perovskite films on different substrates.**

Time-resolved PL decay curves and corresponding fitting curves of perovskite (MAPbI_3_), perovskite/spiro-OMeTAD and perovskite/BP/spiro-OMeTAD films. Samples for PL measurements were prepared by spin-coating perovskite solution, BP dispersion and spiro-OMeTAD solution on quartz substrates.


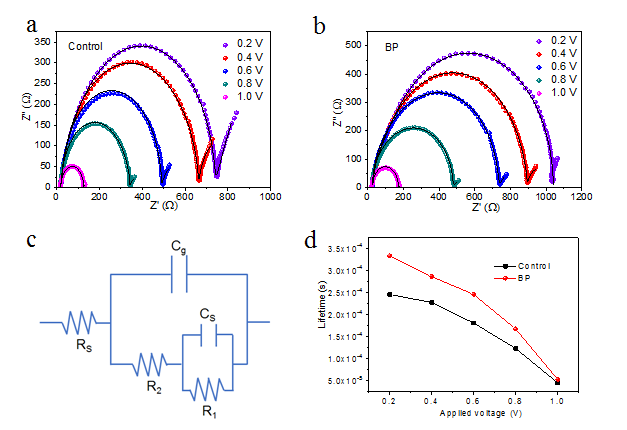


**Fig. S13 | EIS characterization of MAPbI_3_ PSCs.**

**a**,**b**, EIS spectra and fitting curves (black lines) of MAPbI_3_ PSCs without (Control) and with BP deposition (2 times) on perovskite surface at different voltage bias under light illumination (100 mW cm^-2^). **c**, The equivalent circuit used for fitting the experimental data^4^. **d**, Carrier lifetimes as a function of applied voltages derived from the EIS spectra. Note: *C*_g_ and *C*_s_ are high- and low-frequency capacitances of the device, respectively, while *R*_1_ and *R*_2_ represent the low- and high- frequency resistance related to the recombination process. The total recombination resistance is *R_rec_ = R_1_ + R_2_*.


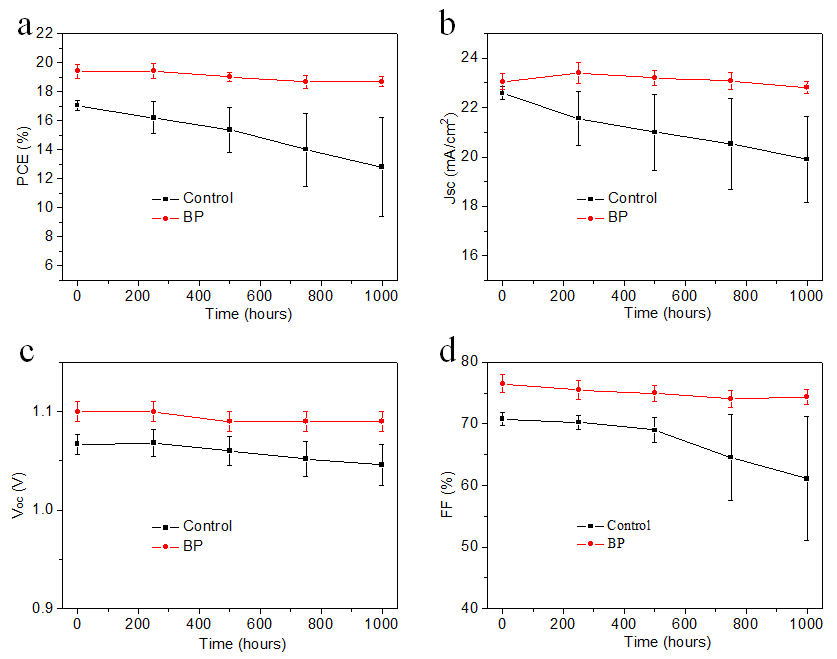


**Fig. S14 | Stability test results of MAPbI_3_ PSCs.**

**a-d**, Evolution of photovoltaic parameters during stability tests of MAPbI_3_ PSCs (average of 10 devices for each condition). All the devices were encapsulated and kept in air with humidity around 30%.

**
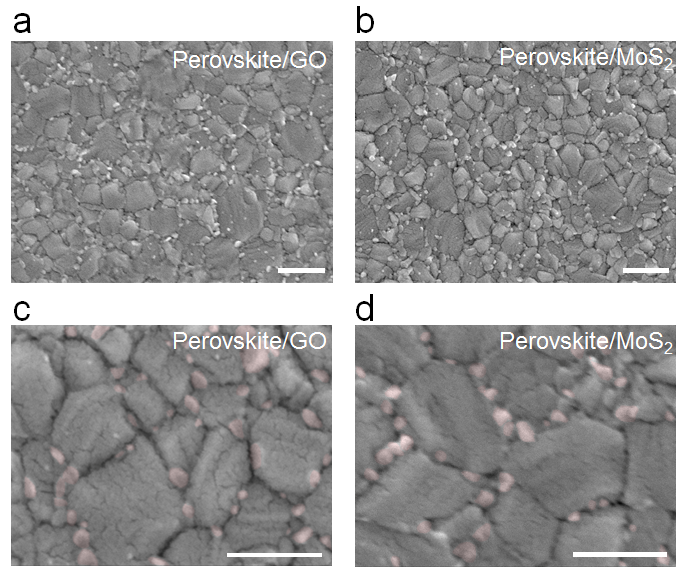
**

**Fig. S15 | Morphology of MAPbI_3_ films modified with GO or MoS_2_.**

**a,b**, Low-magnification scanning electron microscopy (SEM) images of MAPbI_3_ perovskite films modified with GO and MoS_2_ flakes. **c,d**, High-magnification SEM images of MAPbI_3_ perovskite films modified with GO and MoS_2_ flakes. The scale bars are 300 nm.


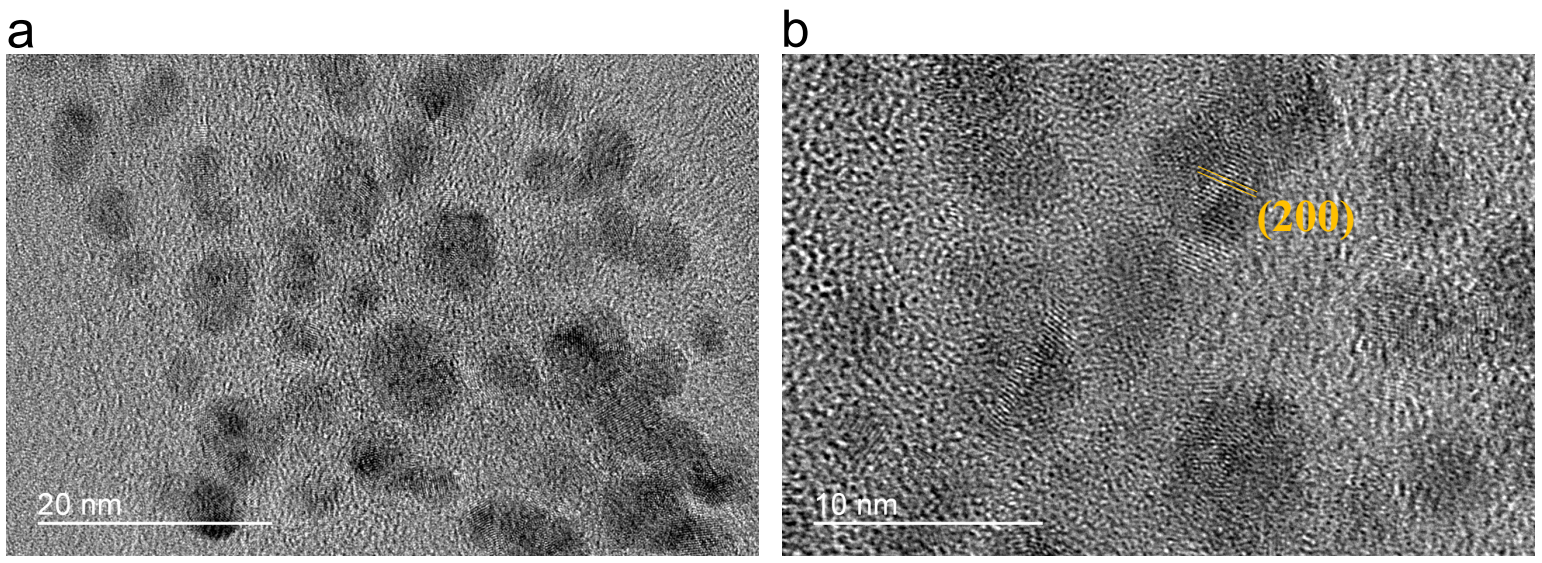


**Fig. S16 | TEM images of BP-QDs with different magnification.**


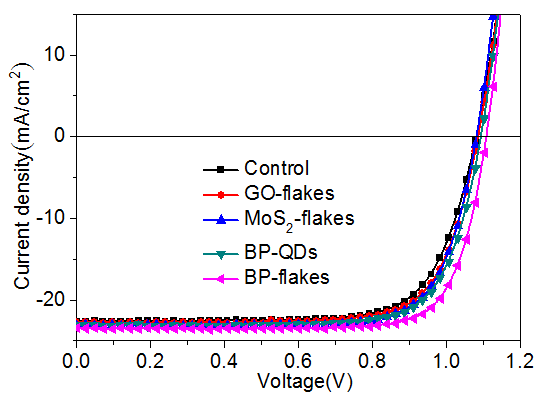


**Fig. S17 | *J-V* curves of MAPbI_3_ PSCs modified with different 2D materials.**

1. **Device simulation of MAPbI_3_ PSCs:**


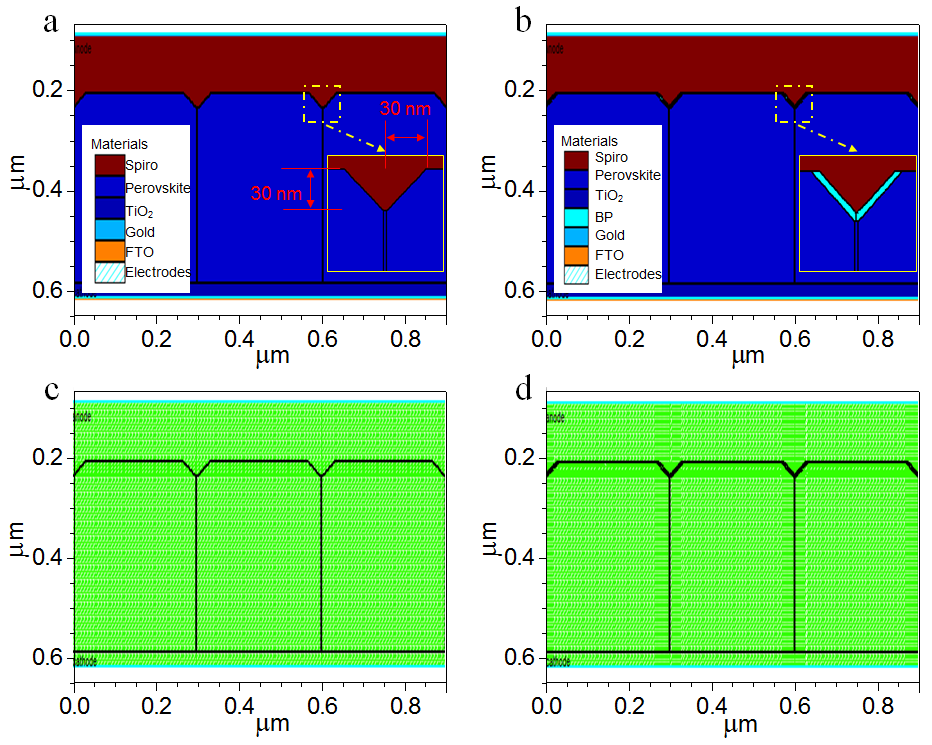


**Fig. S18 | Device structures for simulation of MAPbI_3_ PSCs.**

**a**,**b**, Schematic diagrams of the device structure for simulation. **c**,**d**, The mesh configuration used for the device simulation. The doping level at the GB was optimized to be to fit the experimental *J-V* curves.

Note: Primary Equations Used in Simulation are listed as follows.

Poisson’s Equation:

$\text{div}(\varepsilon\nabla\psi)=-\rho$ (1)

Carrier Continuity Equations:

$\begin{matrix} \frac{\partial n}{\partial t}=\frac{1}{q}div\mathop{J_{n}}\limits^{\to} +G_{n}-R_{n} \\ \frac{\partial p}{\partial t}=\frac{1}{q}div\mathop{J_{p}}\limits^{\to} +G_{p}-R_{p} \end{matrix}$ (2)

Drift-Diffusion:

$\begin{matrix} \mathop{J_{n}}\limits^{\to} =q\mu_{n}n\mathop{E_{n}}\limits^{\to} +qD_{n}\nabla n \\ \mathop{J_{p}}\limits^{\to} =q\mu_{p}p\mathop{E_{n}}\limits^{\to} -qD_{p}\nabla p \end{matrix}$ (3)

Effective Density of States:

$\begin{matrix} n=N_{C}\exp(\frac{E_{F}-E_{C}}{kT_{L}}) \\ p=N_{V}\exp(\frac{E_{V}-E_{F}}{kT_{L}}) \end{matrix}$ (4)

Generation Rate Formula:

$G=\eta_{0}\frac{P\lambda}{hc}\alpha e^{-\alpha y}$ (5)

The doping level of GBs in MAPbI_3_ perovskite film is assumed to be:

Acceptor density = 3.4×10^8^ cm^-2^

The doping level of GBs in mixed perovskite film is assumed to be:

Acceptor density = 2.8×10^8^ cm^-2^

1. **Characterization of mixed perovskite films and PSCs:**


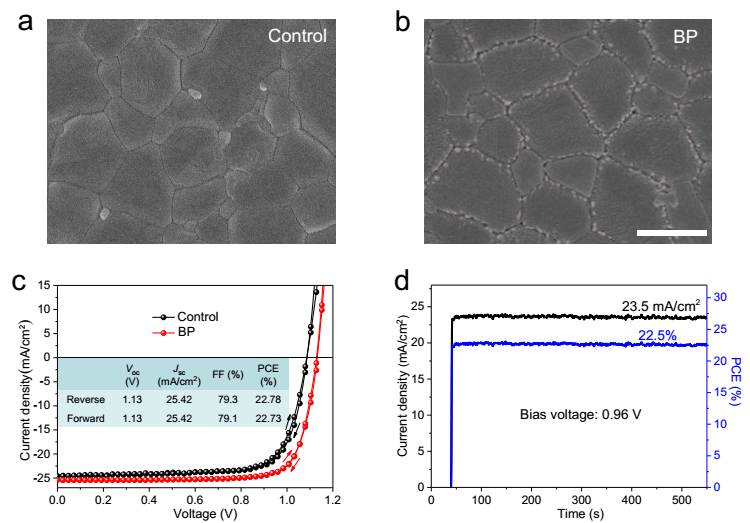


**Fig. S1****9 | Characteristics of mixed perovskite films and devices.**

**a**,**b** SEM images of mixed perovskite films without (Control) and with BP modification (2 times coating). The scale bar is 1 μm. **c**, *J*-*V* curves of the best control and BP-modified devices. **d**, Steady-state photocurrent and efficiency of the champion device measured at the maximum power point.


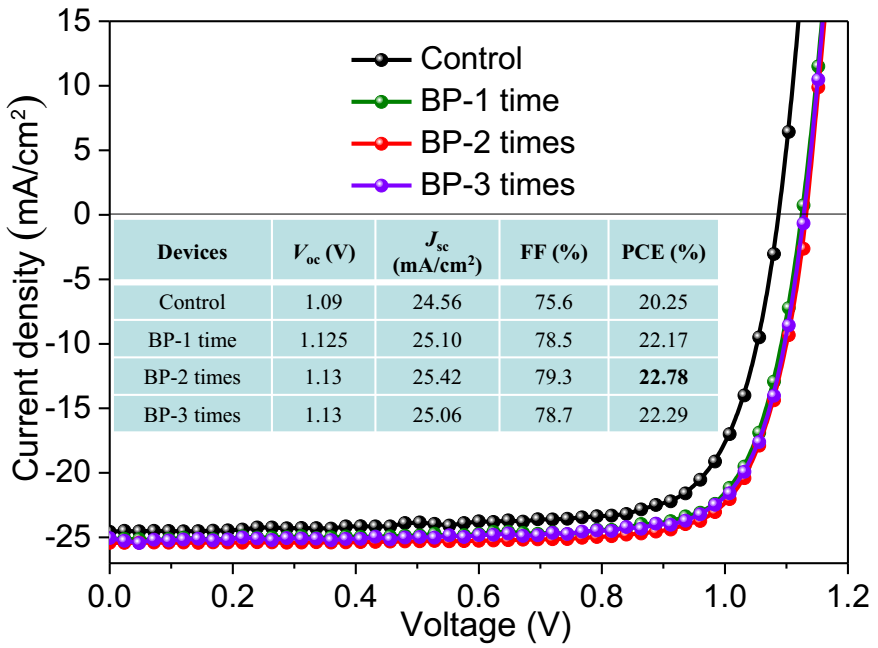


**Fig. S20** **|** *J-V* curves and photovoltaic parameters of mixed PSCs without (Control) and with BP deposition (1~3 times) on perovskite film surface.


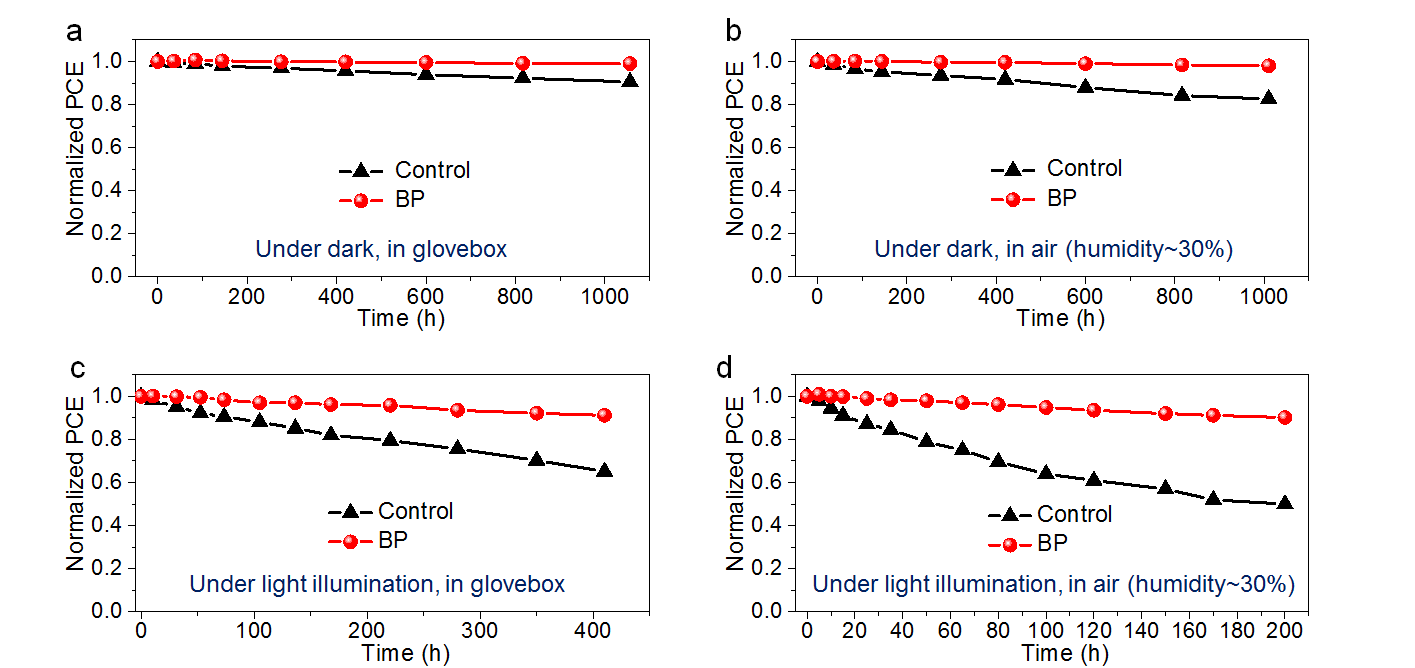


**Fig. S21 | Stability test results of mixed PSCs.**

**a**-**d**, Stability test results of encapsulated mixed PSCs in dark condition or under light illumination (100 mW cm^-2^). Devices for stability test were kept in glovebox or in air (humidity~30%).

1. **Device simulation of mixed PSCs:**


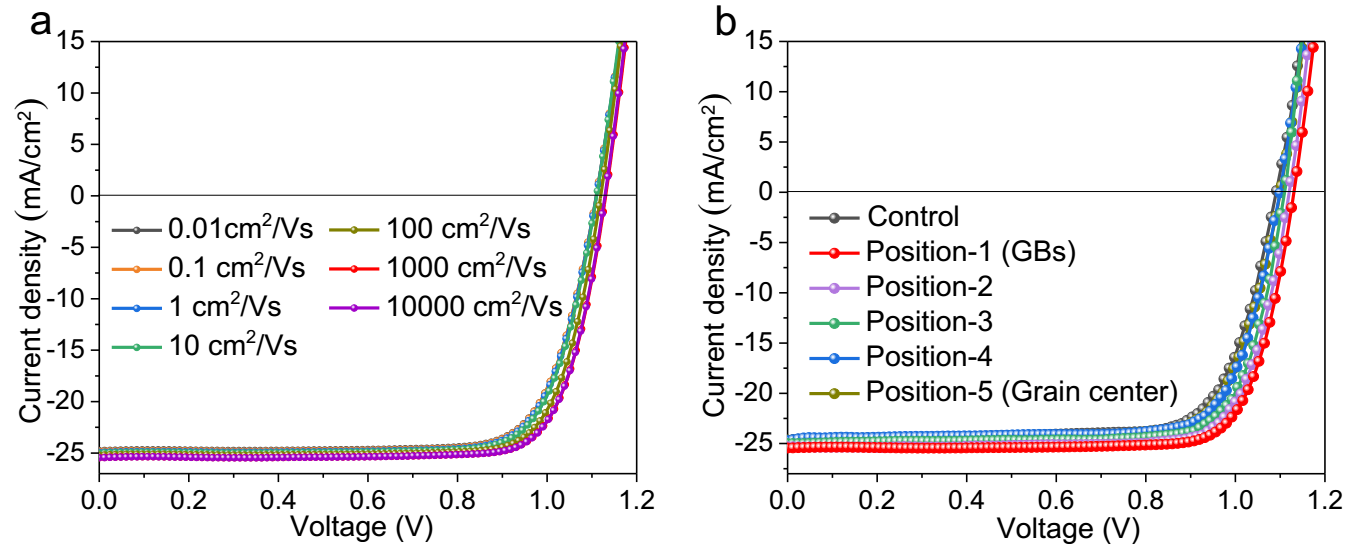


**Fig. S22 | Simulated photovoltaic performance of mixed PSCs.**

**a**, Simulated *J-V* curves of mixed PSCs with varying BP mobility values. **b**, Simulated *J-V* curves of mixed PSCs without (control) and with modification of BP flakes at different positions of perovskite film surface shown in Fig. S23.


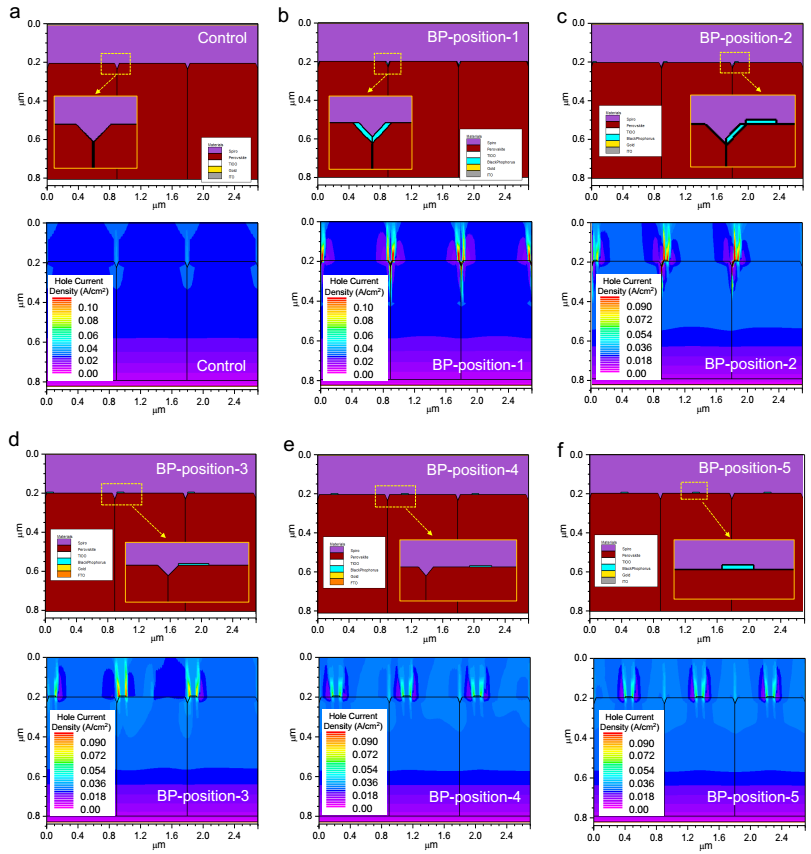


**Fig. S23 | The hole current density distribution in the simulated mixed PSCs. a**-**f**, Device structures and hole current density distribution images in the simulated mixed PSCs, where 2D flakes are located on different positions of a perovskite grain surface.

1. **Supplementary Tables:**

**Table S1 |** Photovoltaic parameters of MAPbI_3_ PSCs without (control) and with BP deposition (1~3 times) on perovskite surface.

| **Devices** | | ***V*_oc_ (V)** | ***J*_sc_ (mA/cm^2^)** | **FF (%)** | **PCE (%)** |
| --- | --- | --- | --- | --- | --- |
| Control | Average | 1.07±0.01 | 22.40±0.50 | 70.8±2.1 | 16.94±0.74 |
|  | Champion | 1.08 | 22.65 | 73.5 | 17.97 |
| BP-1 time | Average | 1.10±0.01 | 22.74±0.21 | 75.7±1.3 | 18.90±0.59 |
|  | Champion | 1.105 | 23.15 | 77.6 | 19.85 |
| BP-2 times | Average | 1.11±0.005 | 22.96±0.19 | 76.8±0.93 | 19.54±0.42 |
|  | Champion | 1.11 | 23.40 | 78.2 | 20.32 |
| BP-3 times | Average | 1.10±0.01 | 22.48±0.31 | 77.5±0.7 | 19.14±0.54 |
|  | Champion | 1.11 | 23.02 | 78.5 | 20.06 |

Note: The experimental value is the average of 30 devices for each condition.

**Table S2 |** Photovoltaic parameters of MAPbI_3_ PSCs without (control) or with BP deposition on perovskite surface (as shown in Fig. S5c), showing *J-V* hysteresis with the forward and reverse scans.

| **Devices** | **Scan direction** | ***V*_oc_ (V)** | ***J*_sc_ (mA/cm^2^)** | **FF (%)** | **PCE (%)** |
| --- | --- | --- | --- | --- | --- |
| Control | Reverse | 1.08 | 22.65 | 73.5 | 17.97 |
|  | Forward | 1.07 | 22.65 | 62.7 | 15.19 |
| BP-2 times | Reverse | 1.11 | 23.40 | 78.2 | 20.32 |
|  | Forward | 1.10 | 23.40 | 75.9 | 19.53 |

**Table S3 |** The average photovoltaic parameters of MAPbI_3_ PSCs with different perovskite grain sizes. The average values were calculated based on 6 devices for each condition.

| **Devices** | | ***V*_oc_ (V)** | ***J*_sc_ (mA/cm^2^)** | **FF (%)** | **PCE (%)** | **PCE Enhancement** |
| --- | --- | --- | --- | --- | --- | --- |
| 200 nm | Control | 1.047±0.010 | 22.27±0.27 | 70.56±0.96 | 16.45±0.34 | - |
|  | BP | 1.090±0.007 | 22.67±0.20 | 72.78±0.90 | 17.98±0.36 | 9.3±2.2 |
| 250 nm | Control | 1.063±0.007 | 22.35±0.19 | 70.81±1.28 | 16.82±0.42 | - |
|  | BP | 1.098±0.007 | 22.87±0.36 | 73.83±1.12 | 18.55±0.44 | 10.3±2.6 |
| 300 nm | Control | 1.073±0.012 | 22.38±0.19 | 71.26±2.18 | 17.08±0.48 | - |
|  | BP | 1.106±0.011 | 23.02±0.17 | 76.52±0.85 | 19.48±0.48 | 14.1±2.8 |
| 390 nm | Control | 1.081±0.008 | 22.44±0.29 | 71.40±1.43 | 17.33±0.53 | - |
|  | BP | 1.109±0.013 | 22.89±0.21 | 75.81±1.28 | 19.24±0.52 | 11.0±3.0 |

**Table S4 |** The photovoltaic parameters of champion MAPbI_3_ PSCs with different perovskite grain sizes.

| **Devices** | | ***V*_oc_ (V)** | ***J*_sc_ (mA/cm^2^)** | **FF (%)** | **PCE (%)** |
| --- | --- | --- | --- | --- | --- |
| 200 nm | Control | 1.05 | 22.33 | 71.8 | 16.84 |
|  | BP | 1.095 | 22.85 | 74.1 | 18.53 |
| 250 nm | Control | 1.07 | 22.46 | 72.1 | 17.33 |
|  | BP | 1.11 | 23.02 | 75.4 | 19.27 |
| 300 nm | Control | 1.085 | 22.55 | 73.2 | 17.91 |
|  | BP | 1.115 | 23.28 | 78.2 | 20.31 |
| 390 nm | Control | 1.095 | 22.61 | 73.7 | 18.26 |
|  | BP | 1.125 | 23.30 | 76.1 | 19.95 |

**Table S5 |** Photovoltaic parameters of MAPbI_3_ PSCs with different interfacial layers, including GO flakes, MoS_2_ flakes, BP QDs and BP flakes (as shown in Fig. S17). Average values are obtained from 30 devices.

| **Devices** | | ***V*_oc_ (V)** | ***J*_sc_ (mA/cm^2^)** | **FF (%)** | **PCE (%)** |
| --- | --- | --- | --- | --- | --- |
| Control | Average | 1.07±0.01 | 22.40±0.50 | 70.8±2.1 | 16.94±0.74 |
|  | Champion | 1.08 | 22.65 | 73.5 | 17.97 |
| GO flakes | Average | 1.075±0.01 | 22.49±0.40 | 73.2±1.5 | 17.68±0.61 |
|  | Champion | 1.085 | 22.72 | 74.8 | 18.45 |
| BP QDs | Average | 1.075±0.01 | 22.76±0.27 | 74.6±1.2 | 18.31±0.50 |
|  | Champion | 1.085 | 23.12 | 75.2 | 18.86 |
| MoS2 flakes | Average | 1.09±0.005 | 22.60±0.25 | 74.8±1.1 | 18.42±0.48 |
|  | Champion | 1.095 | 23.05 | 75.4 | 19.03 |
| BP flakes | Average | 1.11±0.005 | 22.96±0.19 | 76.8±0.93 | 19.54±0.42 |
|  | Champion | 1.11 | 23.40 | 78.2 | 20.32 |

**Table S6 |** Parameters of the perovskite absorber and charge transport layers for device simulation.^1, 5, 17-19^

| **Parameters and units** | **Symbols** | **TiO_2_** | **Perovskite**  **(MAPbI_3_)** | **Perovskite**  **(mixed)** | **Spiro-OMeTAD** |
| --- | --- | --- | --- | --- | --- |
| Thickness (nm) | *L* | 30 | 380 | 600 | 120 |
| Band Gap at 300 K (eV) | *E_g_* | 3.2 | ~1.56 | ~1.51 | 3.0 |
| Electron Affinity (eV) | *χ* | 4.0 | 3.94 | 3.95 | 2.1 |
| Electron density of states at 300 K (#/cm^3^) | *N_C_* | 1×10^21^ | 2.5×10^20^ | 2.5×10^20^ | 2.5×10^20^ |
| Hole density of states at 300 K (#/cm^3^) | *N_V_* | 2×10^20^ | 2.5×10^20^ | 2.5×10^20^ | 2.5×10^20^ |
| Hole mobility (cm^2^ V^-1^ s^-1^) | *μ_h_* | 0.017 | 1.0 | 1.0 | 0.0001 |
| Electron mobility  (cm^2^ V^-1^ s^-1^) | *μ_e_* | 0.017 | 1.0 | 1.0 | 0.0001 |
| Relative dielectric constant | *ε_r_* | 100 | 30 | 46.9 | 3 |
| Doping level (#/cm^3^) | *N_D_* | 2.5×10^17^  (n-type) | - | - | 2.5×10^17^  (p-type) |

Note: The bandgaps of MAPbI_3_ and mixed perovskites are derived from their corresponding EQE curves.

**Table S7 |** Parameters of the 2D materials for device simulation.

| **Parameters and units** | **Symbols** | **BP**^20-22^ | **MoS_2_**^23-25^ | **GO**^16, 26-28^ |
| --- | --- | --- | --- | --- |
| Thickness (nm) | *L* | 5 | 5 | 5 |
| Band Gap at 300 K (eV) | *E_g_* | 1.53 | 1.80 | 1.75 |
| Electron Affinity (eV) | *χ* | 3.79 | 3.40 | 3.42 |
| Electron density of states at 300 K (#/cm^3^) | *N_C_* | 1.04×10^18^ | 9.96×10^18^ | 6.83×10^18^ |
| Hole density of states at 300 K (#/cm^3^) | *N_V_* | 9.16×10^17^ | 7.33×10^18^ | 4.55×10^17^ |
| Hole mobility (cm^2^ V^-1^ s^-1^) | *μ_h_* | 1000 | 73 | 0.015 |
| Electron mobility (cm^2^ V^-1^ s^-1^) | *μ_e_* | 220 | 73 | 0.015 |
| Relative dielectric constant | *ε_r_* | 5.5 | 4 | 15 |

**Table S8 |** Comparison of experimental and simulated photovoltaic parameters of MAPbI_3_ PSCs without (control) and with modification of 2D material on perovskite surface.

| **Devices** | | ***V*_oc_ (V)** | ***J*_sc_ (mA/cm^2^)** | **FF (%)** | **PCE (%)** |
| --- | --- | --- | --- | --- | --- |
| Control | Experimental | 1.08 | 22.65 | 73.5 | 17.97 |
|  | Simulated | 1.072 | 23.23 | 73.2 | 18.24 |
| GO | Experimental | 1.085 | 22.72 | 74.8 | 18.45 |
|  | Simulated | 1.09 | 23.34 | 75.4 | 19.18 |
| MoS_2_ | Experimental | 1.095 | 23.05 | 75.4 | 19.03 |
|  | Simulated | 1.096 | 23.46 | 77.4 | 19.91 |
| BP | Experimental | 1.11 | 23.40 | 78.2 | 20.32 |
|  | Simulated | 1.11 | 23.49 | 78.1 | 20.37 |

**Table S9 |** Hole mobilities of some frequently used hole transport materials in PSCs.

| **HTMs** | Sprio-OMeTAD^5, 6^ | PTAA^7-9^ | PEDOT:PSS^10, 11^ | P3HT  ^6, 12, 13^ | NiO_x_^14^ | CuSCN^15^ | GO^16^ |
| --- | --- | --- | --- | --- | --- | --- | --- |
| **Hole Mobility**  **(cm^2^/Vs)** | 0.0001-0.001 | 0.001-0.01 | ~ 0.01 | 0.0001-0.001 | 0.003-0.01 | 0.01-0.1 | 0.0001-0.015 |

**Table S10 |** Comparison of experimental and simulated photovoltaic parameters of mixed PSCs without (control) and with the modification of 2D materials on perovskite GBs.

| **Devices** | | | ***V*_oc_ (V)** | ***J*_sc_ (mA/cm^2^)** | **FF (%)** | **PCE (%)** |
| --- | --- | --- | --- | --- | --- | --- |
| Control | Experimental | Average | 1.08±0.01 | 24.13±0.56 | 74.0±1.3 | 19.28±0.85 |
|  |  | Champion | 1.09 | 24.56 | 75.6 | 20.25 |
|  | Simulated | | 1.09 | 24.65 | 75.4 | 20.27 |
| GO | Experimental | Average | 1.09±0.01 | 24.64±0.40 | 75.8±0.9 | 20.35±0.62 |
|  |  | Champion | 1.10 | 24.81 | 77.3 | 21.15 |
|  | Simulated | | 1.11 | 24.95 | 77.1 | 21.36 |
| MoS_2_ | Experimental | Average | 1.11±0.005 | 24.80±0.32 | 77.0±0.7 | 21.18±0.48 |
|  |  | Champion | 1.115 | 25.05 | 78.2 | 21.83 |
|  | Simulated | | 1.11 | 25.21 | 78.3 | 21.90 |
| BP | Experimental | Average | 1.125±0.005 | 25.08±0.24 | 78.2±0.6 | 22.05±0.40 |
|  |  | Champion | 1.13 | 25.42 | 79.3 | 22.78 |
|  | Simulated | | 1.13 | 25.45 | 79.3 | 22.81 |

Note: The experimental value is the average of 30 devices for each condition.

**Table S11 |** Simulated photovoltaic parameters of mixed PSCs with varying mobility values of 2D flakes. Here, the 2D flakes are located on perovskite GBs.

| **BP mobility (cm^2^/Vs)** | ***V*_oc_ (V)** | ***J*_sc_ (mA/cm^2^)** | **FF (%)** | **PCE (%)** |
| --- | --- | --- | --- | --- |
| 0.01 | 1.11 | 24.76 | 77.4 | 21.26 |
| 0.1 | 1.11 | 24.83 | 77.3 | 21.30 |
| 1 | 1.11 | 24.93 | 77.5 | 21.45 |
| 3 | 1.11 | 24.94 | 77.7 | 21.50 |
| 10 | 1.11 | 25.01 | 78.1 | 21.67 |
| 30 | 1.11 | 25.15 | 78.5 | 21.92 |
| 100 | 1.12 | 25.21 | 78.9 | 22.28 |
| 300 | 1.12 | 25.44 | 79.0 | 22.51 |
| 600 | 1.12 | 25.45 | 79.8 | 22.74 |
| 1000 | 1.13 | 25.45 | 79.3 | 22.81 |
| 3000 | 1.13 | 25.45 | 79.3 | 22.81 |
| 10000 | 1.13 | 25.46 | 79.3 | 22.82 |

**Table S12 |** Simulated photovoltaic parameters of mixed PSCs with the modification of BP flakes at different positions shown in Fig. S23.

| **Positions of BP flakes** | ***V*_oc_ (V)** | ***J*_sc_ (mA/cm^2^)** | **FF (%)** | **PCE (%)** |
| --- | --- | --- | --- | --- |
| Without BP | 1.09 | 24.65 | 75.4 | 20.27 |
| Position-1 (GBs) | 1.13 | 25.45 | 79.3 | 22.81 |
| Position-2 | 1.12 | 25.31 | 78.9 | 22.37 |
| Position-3 | 1.11 | 25.01 | 77.7 | 21.57 |
| Position-4 | 1.10 | 24.71 | 76.4 | 20.76 |
| Position-5 (Grain center) | 1.10 | 24.67 | 76.1 | 20.66 |

**7. References:**

1. You, P., Liu, Z.K., Tai, Q.D., Liu, S.H. & Yan, F. Efficient Semitransparent Perovskite Solar Cells with Graphene Electrodes. *Adv. Mater.* **27**, 3632-3638 (2015).

2. You, P., Tang, G.Q. & Yan, F. Two-dimensional materials in perovskite solar cells. *Mater. Today Energy* **11**, 128-158 (2019).

3. Tang, G.Q. et al. Solution-Phase Epitaxial Growth of Perovskite Films on 2D Material Flakes for High-Performance Solar Cells. *Adv. Mater.* **31** (2019).

4. Zarazua, I. et al. Operating Mechanisms of Mesoscopic Perovskite Solar Cells through Impedance Spectroscopy and J-V Modeling. *J. Phys. Chem. Lett.* **8**, 6073-6079 (2017).

5. Agarwal, S. & Nair, P.R. Device engineering of perovskite solar cells to achieve near ideal efficiency. *Appl. Phys. Lett.* **107**, 123901 (2015).

6. Kwon, Y.S., Lim, J., Yun, H.-J., Kim, Y.-H. & Park, T. A diketopyrrolopyrrole-containing hole transporting conjugated polymer for use in efficient stable organic–inorganic hybrid solar cells based on a perovskite. *Energy Environ. Sci.* **7**, 1454-1460 (2014).

7. Zhang, W.M. et al. Systematic Improvement in Charge Carrier Mobility of Air Stable Triarylamine Copolymers. *J Am Chem Soc* **131**, 10814-10815 (2009).

8. Heo, J.H. et al. Efficient inorganic–organic hybrid heterojunction solar cells containing perovskite compound and polymeric hole conductors. *Nat. Photonics* **7**, 486-491 (2013).

9. Ryu, S. et al. Voltage output of efficient perovskite solar cells with high open-circuit voltage and fill factor. *Energ. Environ. Sci.* **7**, 2614-2618 (2014).

10. Stavrinidou, E. et al. Direct Measurement of Ion Mobility in a Conducting Polymer. *Adv. Mater.* **25**, 4488-4493 (2013).

11. Rutledge, S.A. & Helmy, A.S. Carrier mobility enhancement in poly(3,4-ethylenedioxythiophene)-poly (styrenesulfonate) having undergone rapid thermal annealing. *J. Appl. Phys.* **114** (2013).

12. Couto, R. et al. Microfluidic supercritical antisolvent continuous processing and direct spray-coating of poly-(3-hexylthiophene) nanoparticles for OFET devices. *Chem. Commun.* **51**, 1008-1011 (2015).

13. Tanase, C., Meijer, E.J., Blom, P.W.M. & de Leeuw, D.M. Unification of the hole transport in polymeric field-effect transistors and light-emitting diodes. *Phys. Rev. Lett.* **91** (2003).

14. Yao, K. et al. A copper-doped nickel oxide bilayer for enhancing efficiency and stability of hysteresis-free inverted mesoporous perovskite solar cells. *Nano Energy* **40**, 155-162 (2017).

15. Pattanasattayavong, P. et al. Electric field-induced hole transport in copper(I) thiocyanate (CuSCN) thin-films processed from solution at room temperature. *Chem. Commun.* **49**, 4154-4156 (2013).

16. Chang, H.X. et al. Thin Film Field-Effect Phototransistors from Bandgap-Tunable, Solution-Processed, Few-Layer Reduced Graphene Oxide Films. *Adv. Mater.* **22**, 4872-4876 (2010).

17. Liu, F. et al. Numerical simulation: Toward the design of high-efficiency planar perovskite solar cells. *Appl. Phys. Lett.* **104** (2014).

18. Nie, W.Y. et al. High-efficiency solution-processed perovskite solar cells with millimeter-scale grains. *Science* **347**, 522-525 (2015).

19. Han, Q.F. et al. Single Crystal Formamidinium Lead Iodide (FAPbI_3_): Insight into the Structural, Optical, and Electrical Properties. *Adv. Mater.* **28**, 2253-2258 (2016).

20. Liu, H., Du, Y.C., Deng, Y.X. & Ye, P.D. Semiconducting black phosphorus: synthesis, transport properties and electronic applications. *Chem. Soc. Rev.* **44**, 2732-2743 (2015).

21. Kumar, P. et al. Thickness and electric-field-dependent polarizability and dielectric constant in phosphorene. *Phys. Rev. B* **93** (2016).

22. Li, L.K. et al. Black phosphorus field-effect transistors. *Nat. Nanotechnol.* **9**, 372-377 (2014).

23. Wang, J.L. et al. High Mobility MoS_2_ Transistor with Low Schottky Barrier Contact by Using Atomic Thick h-BN as a Tunneling Layer. *Adv. Mater.* **28**, 8302-8308 (2016).

24. Kadantsev, E.S. & Hawrylak, P. Electronic structure of a single MoS_2_ monolayer. *Solid State Commun.* **152**, 909-913 (2012).

25. Santos, E.J.G. & Kaxiras, E. Electrically Driven Tuning of the Dielectric Constant in MoS_2_ Layers. *ACS Nano* **7**, 10741-10746 (2013).

26. Guo, R.Y., Li, T. & Shi, S.I. Electron transition pathways of graphene oxide quantum dots unraveled by emission wavelength dependent photoluminescence lifetime. *Rsc Adv.* **7**, 19701-19706 (2017).

27. Liu, Z.K., Lau, S.P. & Yan, F. Functionalized graphene and other two-dimensional materials for photovoltaic devices: device design and processing. *Chem. Soc. Rev.* **44**, 5638-5679 (2015).

28. Nouri, E. et al. Introduction of Graphene Oxide as Buffer Layer in Perovskite Solar Cells and the Promotion of Soluble n-Butyl-substituted Copper Phthalocyanine as Efficient Hole Transporting Material. *Electrochim. Acta* **233**, 36-43 (2017).
